# RNA modulates hnRNPA1A amyloid formation mediated by biomolecular condensates

**DOI:** 10.1038/s41557-024-01467-3

**Published:** 2024-03-12

**Authors:** Chiara Morelli, Lenka Faltova, Umberto Capasso Palmiero, Katarzyna Makasewicz, Marcell Papp, Raphaël P. B. Jacquat, Dorothea Pinotsi, Paolo Arosio

**Affiliations:** 1 Department of Chemistry and Applied Biosciences, Institute for Chemical and Bioengineering, ETH Zurich, Zürich, Switzerland; 2 Scientific Center for Optical and Electron Microscopy, ETH Zurich, Zürich, Switzerland

**Keywords:** RNA-binding proteins, RNA, Intrinsically disordered proteins

## Abstract

Several RNA binding proteins involved in membraneless organelles can form pathological amyloids associated with neurodegenerative diseases, but the mechanisms of how this aggregation is modulated remain elusive. Here we investigate how heterotypic protein–RNA interactions modulate the condensation and the liquid to amyloid transition of hnRNPA1A, a protein involved in amyothropic lateral sclerosis. In the absence of RNA, formation of condensates promotes hnRNPA1A aggregation and fibrils are localized at the interface of the condensates. Addition of RNA modulates the soluble to amyloid transition of hnRNPA1A according to different pathways depending on RNA/protein stoichiometry. At low RNA concentrations, RNA promotes both condensation and amyloid formation, and the catalytic effect of RNA adds to the role of the interface between the dense and dilute phases. At higher RNA concentrations, condensation is suppressed according to re-entrant phase behaviour but formation of hnRNPA1A amyloids is observed over longer incubation times. Our findings show how heterotypic nucleic acid–protein interactions affect the kinetics and molecular pathways of amyloid formation.

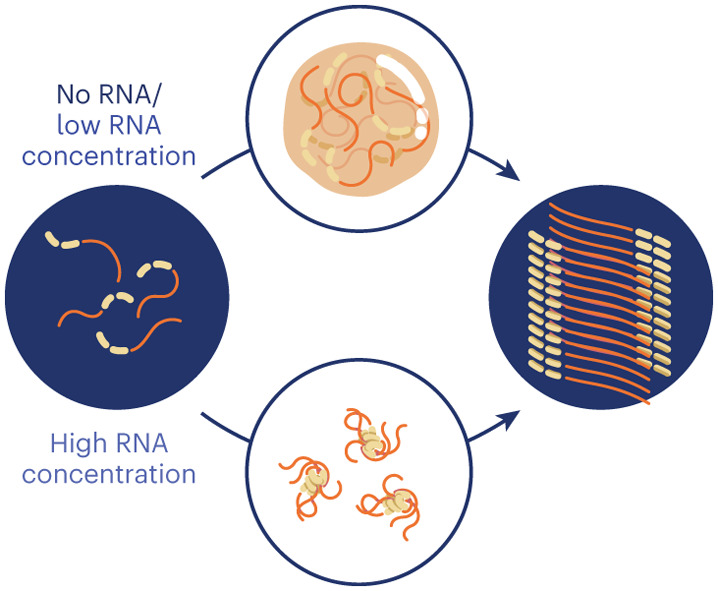

## Abstract (web summary)

The underlying mechanism for how heterotypic protein–RNA interactions modulate the liquid to amyloid transition of hnRNPA1A, a protein involved in amyotrophic lateral sclerosis, has so far remained elusive. Now characterization of hnRNPA1A condensate formation and aggregation in vitro reveals that the RNA/protein stoichiometry affects the molecular pathways leading to amyloid formation.

## Main

Several membraneless organelles formed via the condensation of proteins and nucleic acids contain proteins with prion-like domains that can induce formation of aberrant amyloids over time^1^^,2^. This is the case of stress granules (SGs), membraneless compartments that assemble in the cytoplasm in the presence of environmental stressors. Amyloids associated with amyothropic lateral sclerosis (ALS)^3^ and frontotemporal dementia^4^ are composed of RNA binding proteins (RBPs) that are part of SGs. Moreover, co-localization of fibrils with SGs markers in patients’ tissues^5^^,6^ supports a role of protein condensation in disease onset. Nevertheless, the molecular mechanisms leading to the formation of RBP amyloids within SGs remain unclear.

Multiple disease-associated proteins undergo condensation in vitro, leading to the formation of a dense protein phase surrounded by a dilute phase upon specific modifications in environmental conditions. The resulting dense phase, indicated as biomolecular condensate, is a viscoelastic network with material properties that can change over time^7–12^. The acceleration of amyloid aggregation within condensates is often attributed to the local increase of protein concentration in the dense phase^8^^,13^. However, emerging evidence indicates that other features of condensates can contribute to fibril formation. For instance, amyloid aggregation of hnRNPA1’s low-complexity domain (LCD) is promoted at the interface between the dense and dilute phases^12^.

Another level of complexity in understanding the role of condensates in amyloid aggregation is given by the heterogeneous composition of membraneless organelles^14^^,15^. Heterotypic interactions between the scaffold molecules and aggregation-prone client molecules can have a protective role against amyloid formation despite the high local concentration^16^^,17^, while in other cases they can contribute to the acceleration of amyloid self-assembly^18^. The impact of multicomponent condensates on fibril formation is therefore still unclear. In addition to proteins, SGs are rich in ribonucleic acids, especially messenger RNAs (mRNAs)^19^. It is therefore important to investigate how ribonucleic acids modulate amyloid aggregation mediated by biomolecular condensates.

In protein–RNA systems, the increase of RNA concentration initially promotes the formation of condensates, which eventually is suppressed at higher RNA/protein ratios^20–23^. This phenomenon is known as re-entrant phase behaviour and is explained by the presence of repulsive electrostatic interactions between the negatively charged polymers at high RNA concentration^20^. Re-entrant phase behaviour was suggested as a mechanism exploited by cells to provide feedback control in transcription^23^ and to preserve RBPs’ solubility in the nucleus^24^.

In addition to promoting the formation of condensates at suitable concentrations, ribonucleic acids are often found to accelerate amyloid formation. For instance, aggregation of the tau^25^, prion^26^ and FUS^27^ proteins is favoured by RNA in vitro. Further evidence that RNA may be involved in the formation of fibrils is provided by the cryogenic electron microscopy structures of amyloids extracted from patient tissues, which often present unidentified anionic co-factors at their surface, as in the case of tau in corticobasal degeneration^28^, α-synuclein in Parkinson’s disease^29^ and TDP43 in ALS^30^.

**Fig. 1:**
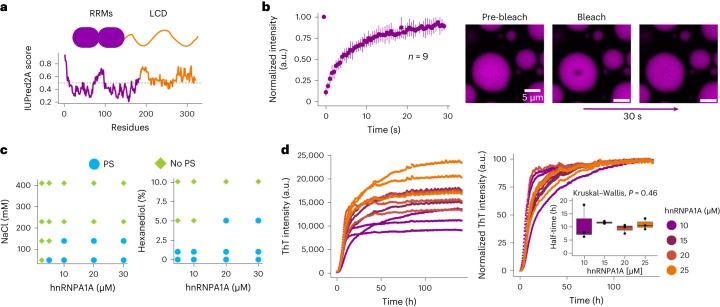
Phase separation mediates hnRNPA1A amyloid formation. **a**, Schematic structure of hnRNPA1A, consisting of the folded RRMs and the disordered LCD, as shown by the online predictor of disordered structures IUPred2A (https://iupred2a.elte.hu)^57^. **b**, (right) FRAP experiments on condensates within 30 min of incubation, showing ~98% signal recovery. Data points represent the mean, and error bars show standard deviations of measurements from nine individual condensates from three technical replicates (*n* = 9). The experiment was repeated for two different protein batches yielding similar results. The hnRNPA1A concentration was 20 μM, and the buffer composition was 20 mM Tris, pH 7.5 and 2 mM β-mercaptoethanol. This buffer was used for all the following experiments unless otherwise stated. (left) Representative confocal microscopy images before and after photobleaching. **c**, The hnRNPA1A phase diagrams as a function of ionic strength and 1,6-hexanediol concentration. PS, phase separation. Phase diagrams were performed with two different protein batches showing similar trends. **d**, Raw and normalized ThT profiles at different protein concentrations. The inset shows a box plot with half-times calculated from normalized curves as the time needed to reach 50% of maximum fluorescence intensity. According to the non-parametric Kruskal–Wallis test (*P* value = 0.46), there is no significant difference between aggregation half-times calculated from reactions with different protein concentrations. This experiment was repeated for three different protein batches yielding similar results; that is, no dependence of half-times on protein bulk concentration was observed. Source data

In this work we aim to understand how RNA affects the phase transitions of hnRNPA1A, an RBP component of the SGs involved in the onset of ALS. hnRNPA1A is a multi-domain protein consisting of a folded domain with two RNA recognition motifs (RRMs) at the N terminus (Fig. 1a; disorder prediction obtained using the IUPred2A tool) and an unstructured C-terminal region that is enriched in G/S-Y/F-G/S motifs and is considered an LCD. This region features arginine-glycine-glycine (RGG) motifs that affect RNA binding, a nuclear localization sequence and several prion-like domains^8,31^. Mutations in the hnRNPA1A LCD are linked to disease onset^32^, and favour fibril formation^32^^,33^. Moreover, the hnRNPA1A LCD drives the maturation of condensates into amyloid fibrils in vitro^8^^,34^.

Here we analyse the self-assembly of hnRNPA1A in vitro using polyuridylic acid (polyU) to mimic unspecific and unstructured cellular RNA molecules, which allows us to contextualize our results with previous experimental and computational work^20,35,36^. In analogy with homotypic condensates of the LCD of hnRNPA1B (see ref. ^12^), we show that the formation of hnRNPA1A amyloids is promoted at the interface of condensates, both with and without polyU. We further demonstrate that in presence of RNA at intermediate concentrations both protein condensation and aggregation are observed. Importantly, we show that higher concentrations of polyU suppress the formation of condensates according to the well-known re-entrant phase behaviour, but do not prevent amyloid formation of hnRNPA1A in bulk over longer timescales. Overall, these results show that RNA can modulate hnRNPA1A phase transitions in a concentration-dependent manner.

## Results

### Condensation mediates hnRNPA1A amyloid aggregation

We analysed the condensation of recombinant full-length hnRNPA1A (biochemical characterization in Supplementary Fig. 1) in 20 mM Tris buffer at pH 7.5 with 2 mM 2-mercaptoethanol. We observed the formation of micrometre-sized condensates above 2.5 μM by bright-field and confocal microscopies (Supplementary Fig. 2). The increase in total protein concentration resulted in higher turbidity, indicating a greater amount of the total dense phase (Supplementary Fig. 3), and induced the formation of larger condensates (Supplementary Fig. 4a–c). However, in the studied concentration range, most of the condensates exhibited a size below 5 μm and similar surface-to-volume ratios (Supplementary Fig. 4d).

The hnRNPA1A condensates were initially dynamic, as shown by the almost complete recovery (approximately 98%) of fluorescence intensity after photobleaching (fluorescence recovery after photobleaching, FRAP; Fig. 1b). Increasing salt or 1,6-hexanediol concentration in the buffer inhibited condensate formation (Fig. 1c and Supplementary Figs. 5 and 6). These results are consistent with the literature^8^ and confirm that different types of interactions contribute to hnRNPA1A condensation.

We next monitored the aggregation of hnRNPA1A in the presence of condensates using thioflavin T (ThT) assay. ThT fluorescence intensity is a common reporter of amyloid formation due to the increase in its quantum yield upon binding to β-sheet structures^37^. The hnRNPA1A aggregation profiles recorded at different protein concentrations are shown in Fig. 1d. The end-point fluorescence intensity increased with protein concentration, indicating that the amount of fibrils formed at the end of the process scales linearly with the total protein concentration. However, the timescale of amyloid formation was independent of the initial protein amount (Fig. 1d). This behaviour, which is consistent with results obtained with the LCD of hnRNPA1B (ref. ^12^), is due to the fact that the increase in protein concentration increases the total volume of the dense phase, but the concentration in the dense phase is determined by the equilibrium with the dilute phase and is independent of the total protein concentration. Across different protein batches, we observed profiles characterized by a steep increase in fluorescence in the first 24 h followed by a slower, steady increase over 5–6 days (Supplementary Fig. 7). The presence of amyloid fibrils at the end of the reaction was confirmed using re-scan confocal microscopy combined with ThT staining (Supplementary Fig. 8).

Importantly, we observed the formation of hnRNPA1A fibrils at high salt concentration (500 mM NaCl), where condensation is suppressed (Supplementary Fig. 9), although with slower kinetics compared to fibril formation mediated by condensation. This result confirms that the formation of condensates and amyloids are interconnected but distinct processes, and that condensates accelerate aggregation but are not required for the formation of hnRNPA1A fibrils.

### Amyloids localize at the interface of hnRNPA1A condensates

**Fig. 2:**
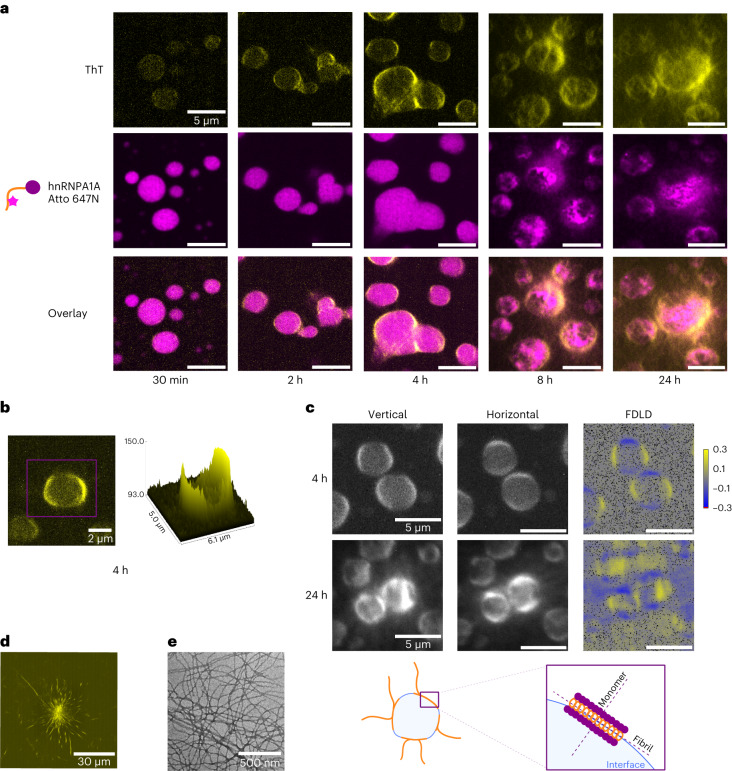
The hnRNPA1A amyloids localize at the interface of the condensates. **a**, Re-scan confocal microscopy images at different time points during hnRNPA1A aggregation mediated by condensation. The protein bulk concentration was 10 μM, and this concentration was used for all the following experiments unless otherwise stated. The top row shows the fluorescence intensity of ThT, reporter for amyloid fibrils. The middle row shows the fluorescence intensity of Atto 647N hnRNPA1A. The bottom row shows an overlay of the ThT and Atto 647N hnRNPA1A fluorescence intensities. Already after 2 h, there is a change in the ThT fluorescence intensity at the interface of the condensates. At later time points, it is possible to notice fibrils emerging from the condensates. This experiment was repeated with at least three protein batches. We always observed a higher ThT signal at the interface of the condensates, but the timescale of fibril formation varied by 3–4 h. **b**, A confocal microscopy image and the corresponding intensity profile showing the strong ThT fluorescence signal at the interface of the condensates after 4 h of incubation. **c**, FDLD imaging reveals a preferential orientation of ThT, indicating that fibrils align parallel to the interface of the condensates. Following an additional 24 h of incubation, the fibrils continue to grow normally towards the interface. ‘Vertical’ and ‘horizontal’ refer to the polarization of the excitation beam. FDLD values are colour-coded in the image from yellow (vertically oriented) to blue (horizontally oriented). Grey values indicate no preferential orientation. The schematic diagram below the microscopy images shows how fibrils and proteins may be oriented with respect to the interface of the condensates. This experiment was independently conducted twice, with each run comprising two technical replicates, using the same protein batch. **d**, Confocal microscopy image of amyloid starbust structure visualized after 4–6 days of incubation, using ThT as the fluorescent reporter. These structures were consistently observed with at least three distinct protein preparations when amyloid aggregation was mediated by condensation. **e**, TEM image of hnRNPA1A amyloid fibrils after 6 days of incubation. TEM imaging of fibrils was performed on at least three different protein batches, yielding fibrils with a similar morphology.

In addition to the temporal evolution of aggregation, we next analysed the spatial formation of fibrils by re-scan confocal microscopy^38^ (Fig. 2a). For these experiments, we tagged hnRNPA1A with the Atto 647N *N*-hydroxysuccinimide (NHS) dye and acquired images within the first 24 h of the aggregation reaction in the presence of ThT. To avoid artefacts due to lysine labelling, the fluorescent protein was diluted 1:300 with non-labelled hnRNPA1A. After 2–4 h we observed the formation of a ThT positive rim at the condensate surface (Fig. 2b and Supplementary Fig. 10). In another set of experiments, we stained hnRNPA1A condensates with different extrinsic dyes. Importantly, we observed an increase in fluorescence signal at the droplet interface only with extrinsic dyes that are known reporters of amyloid fibrils, such as ThT and Amytracker 680, while the fluorescence signal of the rhodamine and Atto 647N NHS dyes was uniform within the condensates (Supplementary Fig. 11).

**Extended Data Fig. 1:**
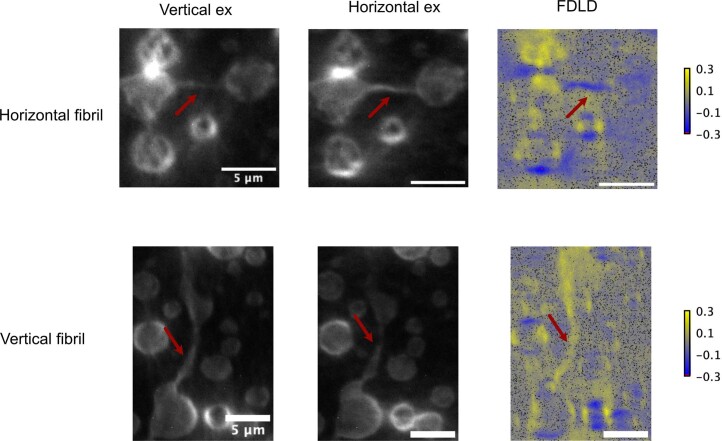
hnRNPA1A amyloid fibrils are oriented parallel to the interface of the condensates. To further prove the orientation of fibrils at the interface of the condensates, we applied Fluorescence Detected Linear Dichroism (FDLD) in combination with ThT staining to analyse amyloid fibrils formed at later incubation times (24-48 h). From the images, it is possible to observe that ThT molecules bound to horizontally oriented fibrils are preferentially excited by horizontally polarized light (color-coded in blue in the FDLD image, see Material and Methods). In contrast, ThT molecules bound to vertically oriented fibrils are preferentially excited by vertically polarized light (color-coded in yellow in the FDLD image). This experiment was performed in technical duplicates for one single protein batch.

To obtain further information about the arrangement of fibrils at the interface of the condensates, we applied fluorescence-detected linear dichroism (FDLD) imaging^39^. By modulating the polarization of the incident laser beam in a confocal microscope, this technique enables the measurement of fluorophore emission intensity after sequential excitation with vertically and horizontally polarized light. According to the photoselection principle, the probability of photon absorption is higher when the dye’s transition dipole aligns parallel to the excitation light. Therefore, variations in emission intensity due to laser polarization provide information about the orientation of fluorophores. When combined with ThT staining, FDLD imaging can be applied to investigate the arrangement of amyloid fibrils, since ThT binds to fibrils with its transition dipole oriented parallel to their long axis^40^^,41^. Briefly, during data acquisition, the sample is sequentially excited with vertically and horizontally polarized light. Afterwards, FDLD values are calculated pixel by pixel as the ratio between the sum and difference of the emission intensities after sample excitation with vertically and horizontally polarized light. Using this method, we observed a preferential orientation of amyloids parallel to the surface of the condensates (Fig. 2c and Extended Data Fig. 1). At longer timescales, fibrils also propagated normally to the interface. As a control, we repeated the experiments with model condensates made of other scaffold proteins^42^ and polymers^43^ that do not undergo aggregation. No preferential orientation of ThT was observed at the interface of these condensates, proving that this effect is related to fibril formation (Supplementary Figure 12).

At longer incubation times, re-scan confocal microscopy revealed the appearance of star-shaped aggregates (Fig. 2d). Moreover, we confirmed the presence of amyloid fibrils using transmission electron microscopy (TEM; Fig. 2e).

### PolyU modulates hnRNPA1A condensation

After analysing the formation of amyloid fibrils in homotypic condensates, we investigated how polyU modulates hnRNPA1A phase transitions. We used polyU molecules with a molecular weight distribution between 800 and 1,000 kDa; in the following, we report polyU concentration in nanograms per microlitre. For stoichiometry and charge calculations, we considered an average molecular weight of 900 kDa (details on calculations in Supplementary Table 1).

**Fig. 3:**
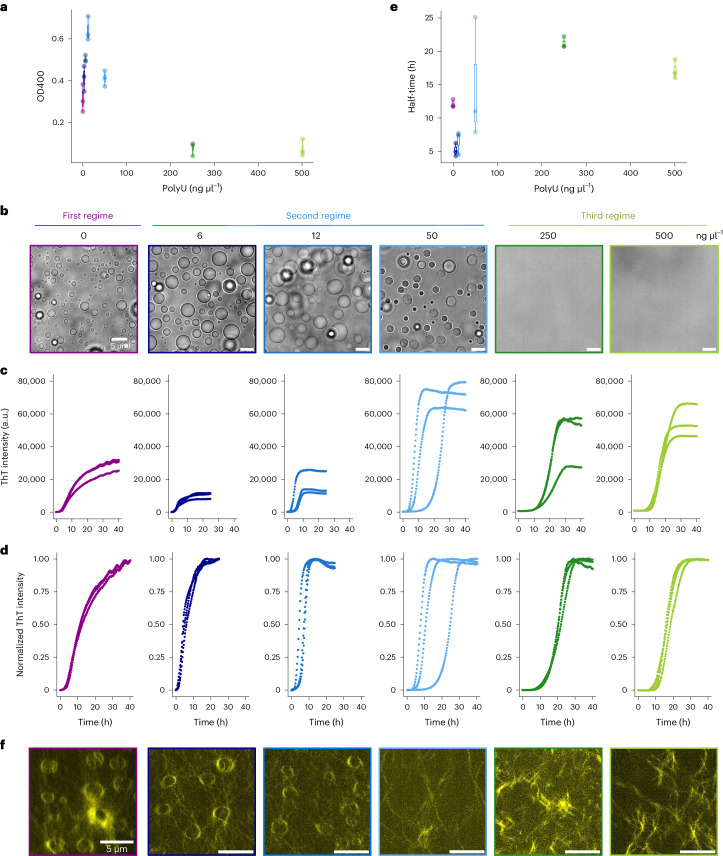
PolyU modulates hnRNPA1A condensation and amyloid formation in a concentration-dependent manner. **a**, The change in volume of the hnRNPA1A dense phase at a fixed bulk concentration as measured by turbidity (OD400, the optical density at 400 nm) in the presence of increasing polyU concentrations. Turbidity measurements were performed in technical triplicates (*n* = 3) and with two distinct protein preparations, yielding similar results. **b**, Bright-field microscopy images showing the absence and presence of protein condensates at different polyU concentrations and constant protein concentration after 30 min of incubation. Accordingly, we delineate three different regimes: (1) condensation driven by homotypic interactions; (2) condensation driven by both homotypic and heterotypic protein–RNA interactions; and (3) re-entrant phase and absence of condensation. The regimes are colour-coded in all figures by purple, blue and green colours, respectively. Bright-field imaging was always performed in parallel with turbidity measurements to confirm the presence of condensates, and was therefore repeated for two different protein preparations. **c**,**d**, Raw and normalized ThT profiles of hnRNPA1A at increasing concentrations of polyU. The experiment was performed with three protein batches, each with three technical replicates. **e**, Half-times of hnRNPA1A aggregation kinetics calculated from the normalized curves shown in **d** as a function of polyU concentration (*n* = 3). **f**, Re-scan confocal microscopy images of end-point samples (48 h), using ThT as fluorophore. Fibrils are rearranged in a circular shape resembling the structure of the condensates only in the absence or at a very low concentration of polyU. Confocal imaging was performed for each condition at the end of the aggregation reaction to visualize fibrils, and was repeated for two different protein preparations. Source data

We first analysed the effect of polyU on hnRNPA1A condensation during a short incubation time. At constant protein concentration (10 μM), we measured the turbidity of mixtures containing increasing RNA concentrations and observed a maximum at 12 ng μl^–1^ of polyU (Fig. 3a). Bright-field microscopy analysis showed the formation of hnRNPA1A condensates up to a polyU concentration of 50 ng μl^–1^ (Fig. 3b), which corresponds approximately to a protein/RNA molar ratio of 180 and charge ratio of ~0.4. At higher RNA concentrations (250 ng μl^–1^ and 500 ng μl^–1^), we did not observe any micrometre-sized condensates.

Overall, we could distinguish three different regimes according to polyU concentration: (1) formation of micrometre-sized condensates mediated by homotypic protein–protein interactions in the absence of polyU, (2) formation of micrometre-sized condensates favoured by protein–nucleic acid interactions in addition to protein–protein interactions at low to intermediate polyU concentrations and (3) absence of condensation in the presence of heterotypic protein–nucleic acid interactions at high polyU concentration. These three regimes are represented in Fig. 3b and are colour coded (as purple, blue and green, respectively) in all figures.

### Effect of polyU on hnRNPA1A amyloid aggregation

**Extended Data Fig. 2:**
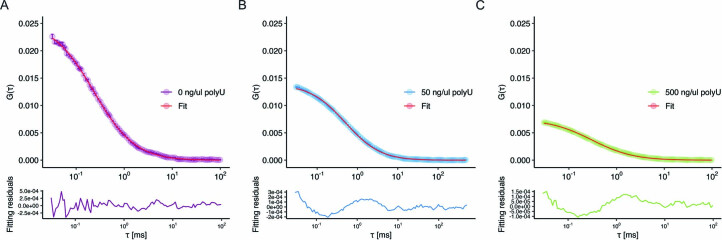
Measurement of monomer conversion using Fluorescence Correlation Spectroscopy (FCS). Representative auto-correlation curves acquired on atto647N hnRNPA1A after 72h of incubation with (A) 0 ng/ul polyU (B) 50 ng/ul polyU and (C) 500 ng/ul polyU. The curves were fitted with a model assuming a single diffusing component. Data points represent the mean and error bars standard deviation of the measurement. The fitting residuals are showed below each graph. The amplitude decrease of the auto-correlation curve with increasing polyU concentration indicates that RNA contributes to protein solubility. hnRNPA1A concentration calculated in each condition was 1.68 ± 0.51 uM in absence of polyU, 2.5 ± 0.18 uM with 50 ng/ul uM polyU and 4.68 ± 0.20 uM.

Next, we investigated the effect of increasing polyU concentration on hnRNPA1A fibril formation using ThT assay, after verifying that the presence of polyU does not cause an increase in the ThT fluorescence signal over time (Supplementary Fig. 13) and that polyU alone does not form condensates in the conditions tested (Supplementary Fig. 14). As shown in Fig. 3d, RNA greatly affects hnRNPA1A aggregation kinetics. The sigmoidal shape of the curves shows that, in the presence of polyU, the ThT profile reaches a plateau. By contrast, in the absence of polyU, the ThT fluorescence signal continues to increase gradually. To compare the overall aggregation rates, we calculated the half-time of each of the normalized curves as the time needed to reach half of the maximum fluorescence intensity (Fig. 3c–e). The shortest half-time was observed in the second regime, where both RNA and condensates are present. In addition to the kinetic profiles, we evaluated the monomer concentration in the dilute phase after 72 h incubation, when the ThT values reached a plateau, using fluorescence correlation spectroscopy (FCS; Extended Data Fig. 2). The monomer conversion was higher in the presence of condensates (83 ± 5% and 75 ± 2% in the absence and presence of 50 ng μl^–1^ polyU, respectively) with respect to the re-entrant phase condition (53 ± 2% in the presence of 500 ng μl^–1^ polyU).

At the end of the incubation, we analysed the amyloid fibrils of each condition with confocal microscopy by recording the ThT signal (Fig. 3f). In the first regime and in part of the second regime, the fibrils were arranged in a circular shape that is reminiscent of condensates. Such organization was not observed in the third regime.

**Fig. 4:**
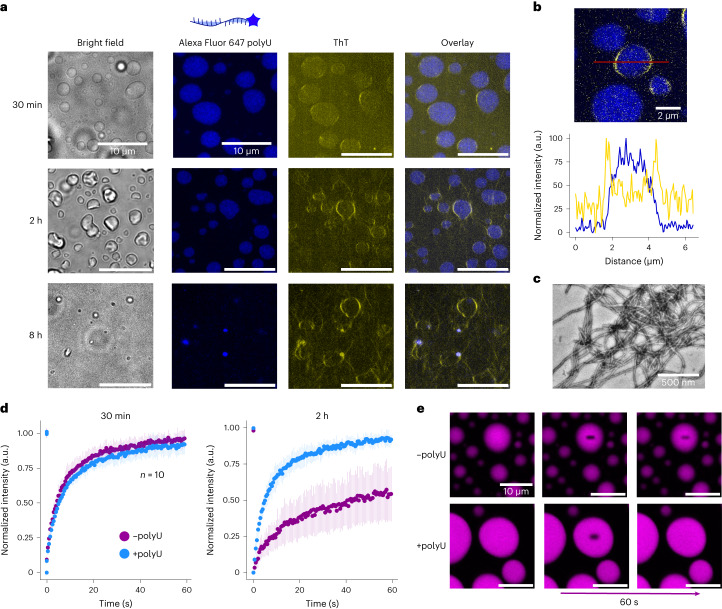
Fibril formation within heterotypic condensates. **a**, Bright-field and re-scan confocal images of hnRNPA1A condensates in the presence of 50 ng μl^–1^ polyU labelled with Alexa Fluor 647 Hydrazide. This condition was taken as representative of the second regime, colour-coded with blue in Fig. 3. Already after 30 min, we observe a higher ThT signal at the interface of heterotypic condensates. In this condition, condensates dissolve completely or partially, and within a few hours are replaced by amyloid fibrils. Imaging of hnRNPA1A aggregation in the presence of labelled polyU was performed with two distinct protein preparations. The time of condensate dissolution varied by a few hours depending on the protein batch. **b**, Confocal image and corresponding intensity profiles showing a higher ThT signal at the interface of hnRNPA1A condensates after 2 h of incubation, while labelled polyU is uniformly distributed. **c**, TEM image of hnRNPA1A amyloid fibrils in the reference condition of the second regime after 72 h of incubation. TEM analysis of hnRNPA1A fibrils formed in the second regime was conducted with at least three distinct protein preparations. **d**, FRAP experiments on homotypic (–polyU) and heterotypic (+polyU) condensates after incubation for 30 min and 2 h. After 30 min, condensates both without and with RNA show high fluorescence recovery after photobleaching (±95%). After 2 h, the fluorescence recovery remains constant for heterotypic condensates, while it decreases for homotypic condensates. Protein and polyU concentrations were 20 μM and 100 ng μl^–1^, respectively (corresponding to the protein/RNA ratio of the second regime). FRAP experiments were performed in technical duplicates with two distinct protein preparations, and for each condition the recovery time after photobleaching was measured from *n* = 10 different droplets. The graph reports the average of ten measurements, and error bars show the standard deviation. **e**, Examples of confocal microscopy images of the FRAP experiments, showing the difference in FRAP between homotypic and heterotypic condensates after 2 h of incubation. Source data

We characterized the spatial evolution of amyloids within droplets in the second regime using as a reference condition a polyU concentration of 50 ng μl^–1^. To this purpose, we used ThT as a reporter of fibrils and we labelled polyU with Alexa Fluor 647 Hydrazide to follow its localization in condensates over time. We observed that polyU partitions into the condensates, and starting from 30 min of incubation, there is a higher ThT intensity at the condensate surface (Fig. 4a,b). At 2 h there is an increasing formation of fibrils and drastic changes in condensate morphology (Fig. 4a). At longer incubation times, condensates partially or completely dissolve (Fig. 4a). The presence of amyloids at the end of the reaction was further confirmed by TEM analysis (Fig. 4c).

Condensate ageing is often associated with the formation of intermolecular β-sheet structures and/or loss of liquid-like properties^36^. To test the effect of polyU on the ageing of hnRNPA1A condensates, we performed FRAP experiments at 30 min and 2 h of incubation. We monitored the fluorescent signal of hnRNPA1A labelled with Atto 565 NHS, diluted 1:20 with unlabelled protein. Shortly after droplet formation, the FRAP values of hnRNPA1A in homotypic and heterotypic condensates are comparable (96 ± 6% and 91 ± 7% fluorescence recovery, respectively). However, after 2 h the fluorescence recovery greatly decreases in homotypic condensates (54 ± 19%), while it remains constant in heterotypic condensates (91 ± 3%).

**Fig. 5:**
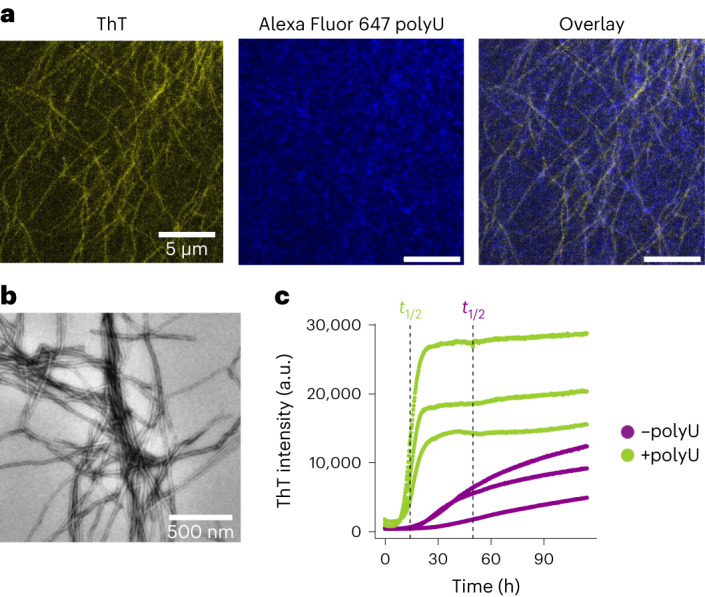
PolyU promotes hnRNPA1A amyloid formation in the absence of condensation. **a**, Re-scan confocal fluorescence microscopy images of hnRNPA1A fibrils after 48 h of incubation with 500 ng μl^–1^ polyU. This condition was taken as representative of the third regime in Fig. 3. Imaging of fibrils with re-scan microscopy in the third regime was repeated with at least two distinct protein preparations. **b**, TEM image of hnRNPA1A amyloid fibrils in the third regime after 48 h. TEM analysis was conducted on at least two distinct protein preparations. **c**, ThT aggregation profiles of hnRNPA1A with 500 mM NaCl (in the absence of protein condensation) in the absence (–) and presence (+) of 500 ng μl^–1^ polyU. The dashed vertical lines show the half-times (*t*_1/2_). The aggregation assays were performed in technical triplicates and with two distinct protein preparations. Source data

At 500 ng μl^–1^ polyU (third regime), we could visualize fibrils at the end of the reaction with re-scan confocal microscopy and TEM (Fig. 5a,b).

To test whether polyU accelerates amyloid formation in the absence of condensation, we prepared solutions of hnRNPA1A in the absence and presence of 500 ng μl^–1^ polyU at 500 mM NaCl, where condensate formation is suppressed (Supplementary Figs. 5 and 9). Remarkably, in this condition polyU accelerated the formation of amyloids, reducing the half-time from 50 h to 16 h (Fig. 5c).

### Testing RNAs of different lengths and sequences

**Extended Data Fig. 3:**
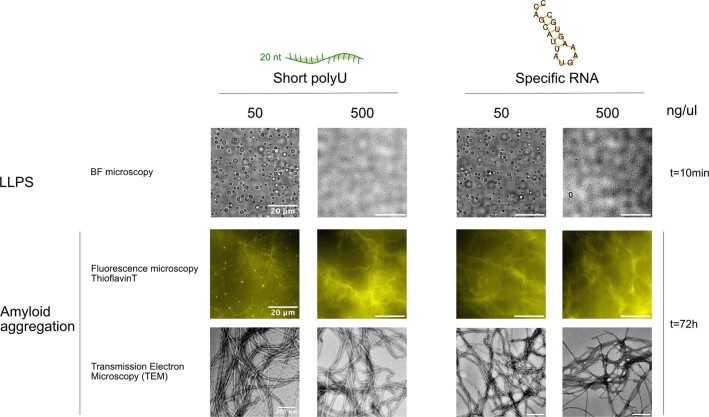
Different RNAs modulate re-entrant phase behavior and amyloid formation of hnRNPA1A in a similar manner. Top row shows bright field microscopy images of hnRNPA1A solutions at 10 uM bulk concentration in presence of 50 or 500 ng/u l of short polyU or specific RNA. For both types of RNA we observe formation of condensates only at the lower concentration, consistent with the re-entrant phase behaviour observed with long polyU. The second and third rows show, respectively, fluorescence microscopy and TEM micrographs of samples after 72 h incubation and demonstrate the presence of amyloid fibrils. The experiments were performed on two independent protein preparations yielding similar results. The secondary structure of the specific RNA was predicted using RNAfold WebServer, (http://rna.tbi.univie.ac.at/cgi-bin/RNAWebSuite/RNAfold.cgi).

Finally, we investigated whether the observed effects are shared by other types of RNA molecules. To this aim, we analysed the effect of a shorter polyU molecule (20 nt, indicated in the following as ‘short polyU’) and an RNA molecule featuring the sequence specifically recognized by hnRNPA1A RRMs^44^. This sequence features 18 nucleotides (5′ CC AGC AUU AUG AAA GUG C 3′) and its secondary structure is shown in Extended Data Fig. 3 (secondary structure obtained using RNAfold web server^45^). In the following text, this RNA molecule is indicated as ‘specific RNA’.

We applied bright-field microscopy to observe the formation of condensates, and fluorescence microscopy combined with TEM to assess the presence of amyloid fibrils after 72 h of incubation. As reference conditions, we analysed 10 μM hnRNPA1A in the presence of 50 or 500 ng μl^–1^ of RNA, which correspond to the protein/RNA ratios of the second or third regime, respectively.

As seen in Extended Data Fig. 3, both the short polyU and the specific RNA suppress hnRNPA1A condensation at the higher concentration, similarly to the long polyU. Moreover, after 72 h of incubation, we observed formation of amyloid fibrils in all conditions tested. Fibrils do not exhibit evident changes in their mesoscopic morphology according to RNA length and sequence. The kinetics of fibril formation were similar for short and long polyU (Supplementary Fig. 15). ThT profiles in the presence of specific RNA could not be recorded due to a high ThT fluorescence signal in the control samples.

## Discussion

In this work we investigated how condensation and protein–RNA heterotypic interactions mediate the formation of hnRNPA1A amyloid fibrils.

In the absence of RNA, we observed that full-length hnRNPA1A forms dynamic condensates that over time convert into amyloid fibrils (Fig. 2). Condensate formation is suppressed at physiological ionic strength (Fig. 1c), while aggregation is observed at both low and high salt concentrations (Fig. 1d and Supplementary Fig. 9). Our results confirm that condensation and fibril formation are driven by different types of intermolecular interactions^8^.

ThT kinetic profiles of hnRNPA1A aggregation at low salt concentration in the absence of RNA present a steep increase in fluorescence during the first 24 h of incubation followed by a slower increase in signal over time (Fig. 1d and Supplementary Fig. 7). Similar profiles were observed for condensation-mediated aggregation of the LCD of TDP43 (ref. ^46^), and may be explained by different aggregation rates in the dense and dilute phases. Moreover, in our system, the slow increase in ThT fluorescence can also be due to the emergence of the ‘starburst’ structures observed at later incubation times (Fig. 2d).

We have previously shown that for the LCD of hnRNPA1B, fibril formation is promoted at the interface of the condensates^12^. Importantly, here we demonstrate that this mechanism occurs also in condensates of full-length hnRNPA1A, as revealed by confocal microscopy using ThT and Amytracker 680 for staining (Fig. 2 and Supplementary Fig. 11). Moreover, with FDLD imaging, we have shown that fibrils are aligned parallel to the interface of the condensates, indicating that monomers are oriented normally to the surface (Fig. 2c). This result is consistent with molecular dynamic simulations showing that condensates of the hnRNPA1A LCD exhibit a networked structure in which the conformation and orientation of the protein at the interface differ from in the bulk, potentially exposing aggregation-prone regions and promoting aggregation^47^. Experimentally, surface-mediated phase transitions were reported for other RBPs involved in ALS including FUS^48^^,49^ and the LCD of TDP43 (ref. ^50^), supporting the notion that this may be a general mechanism for amyloid aggregation mediated by condensation.

After characterizing the liquid to solid transition in homotypic hnRNPA1A condensates, we progressively increased RNA concentrations to modulate the balance between protein–protein and protein–RNA interactions (Fig. 3).

Low polyU concentrations (second regime) promote condensation, in agreement with previous experimental evidence^8^ and computational analysis^35^. PolyU partitions with hnRNPA1A condensates (Fig. 4a) and increases the dense phase volume (Fig. 4b). According to the computational study of Tejedor et al.^35^, heterotypic interactions between hnRNPA1A and polyU are multivalent and mainly of an electrostatic nature. While the highest number of contacts was observed between the LCD and polyU, the strongest interactions occur between RNA and the arginine and lysine residues of the RRMs.

Despite exhibiting a similar monomer conversion (83 ± 5% and 75 ± 2%, respectively; Extended Data Fig. 2), in this regime, aggregation proceeded faster compared to the homotypic system (Fig. 3). With re-scan confocal microscopy, we observed that also in presence of polyU, fibrils localize at the interface of the condensates (Fig. 4a,b). However, with the heterotypic condensates, the emergence of the ThT positive rim was faster compared to the first regime, and over time, condensates partially or fully dissolved within a few hours of incubation (Fig. 4a). Dissolution of the condensates is likely due to the decrease of protein concentration within the dense phase below the concentration required for formation of condensates, as a consequence of the recruitment of the monomer into amyloid fibrils. This phenomenon was not observed in the absence of polyU, possibly due to a competition between the formation of amyloids and ageing of the condensates towards a gel-like material. This mechanism is consistent with the FRAP experiments showing that the mobility of hnRNPA1A within homotypic condensates is greatly reduced over time, while it remains constant in heterotypic droplets (Fig. 4d,e). Moreover, the higher mobility of hnRNPA1A in the presence of polyU may contribute to the accelerated aggregation in the second regime, since it leads to faster diffusion towards the interface of the condensates, where fibril formation is favoured.

Higher polyU concentrations inhibit condensate formation (Fig. 3a,b), as previously reported with other proteins and RBPs^20,24,35^. We observed this well-known re-entrant phase behaviour at RNA/protein ratios larger than 0.7, consistent with the literature^24^. However, despite the absence of condensation in this regime, we still observed fibril formation over time, as revealed by ThT staining, confocal microscopy and TEM (Figs. 3f and 5).

Moreover, in conditions where condensation is suppressed, high polyU concentrations accelerated hnRNPA1A aggregation (Fig. 5c). Poly-anion-induced aggregation has been observed for a plethora of aggregation-prone proteins including tau^51^, α-synuclein^52^^,53^, Aβ (ref. ^54^) and FUS^27^. The exact molecular mechanisms behind hnRNPA1A-accelerated aggregation in the presence of RNA requires further analysis. PolyU might favour the formation of fibrils by inducing a local increase in hnRNPA1A concentration. Also, preferential RRM–RNA binding might result in protein conformational changes that favour the formation of intermolecular contacts between aggregation-prone regions^44,55^. To understand this phenomenon, it will be crucial to investigate the binding mode and interaction strength between RNA and hnRNPA1A monomers and fibrils^56^, in both the dense and the dilute phases.

**Fig. 6:**
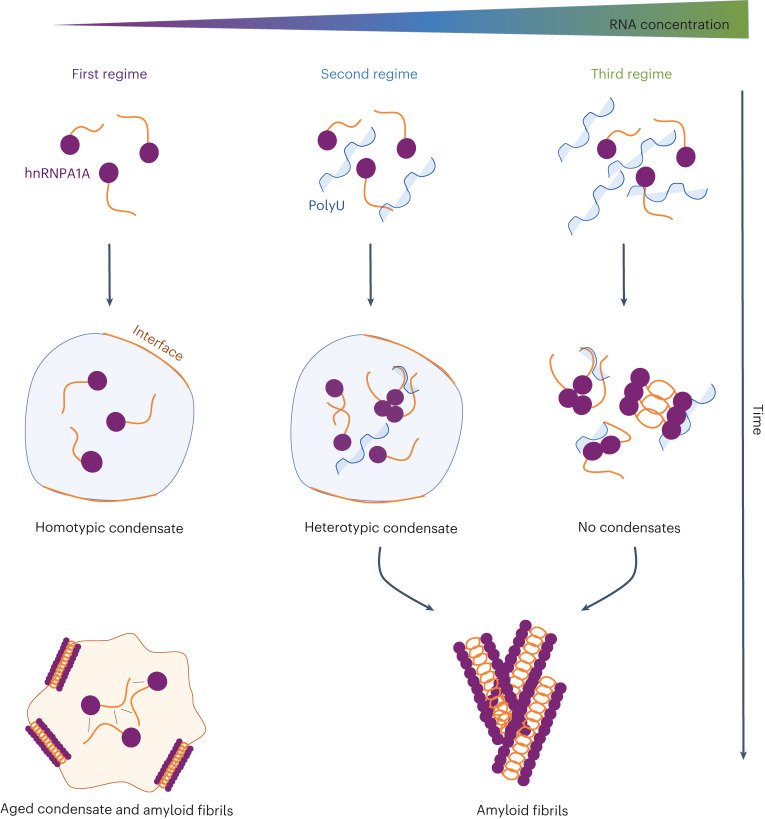
Concentration-dependent polyU mediation of hnRNPA1A condensation and amyloid formation. Schematic representation of the three regimes observed with the different RNA concentrations. In the absence of RNA, formation of amyloid fibrils proceeds via condensation, and aggregation is promoted at the interface of the condensates. In the second regime, at low RNA concentration, heterotypic protein–RNA interactions promote condensation and fibril formation, which also in this case are observed at the interface of the condensates. In the third regime, at high RNA concentration, condensation is suppressed, and the formation of amyloid fibrils occurs without condensation. Bright-field and fluorescence microscopy images of the time evolution of amyloid formation are shown in Supplementary Figs. 16–18.

Our analysis demonstrates how RNA can modulate full-length hnRNPA1A phase transitions. In the absence and at low concentrations of RNA, fibril formation proceeds via a two-step process mediated by condensation, in which hnRNPA1A initially forms dynamic, liquid-like condensates that age over time into amyloid fibrils (Supplementary Fig. 16). Amyloid formation is promoted at the interface of the condensates, where fibrils align parallel to the surface. At low concentrations, the presence of RNA provides a further catalytic effect, which accelerates fibril formation while simultaneously promoting condensation (Supplementary Fig. 17). Higher RNA concentrations suppress condensation, but fibril formation is observed over longer incubation time (Supplementary Fig. 18). This mechanism is schematized in Fig. 6 and was shared by the different RNA molecules tested in this work (Extended Data Fig. 3 and Supplementary Fig. 15), suggesting a generic effect, although future analysis with additional RNA sequences and structures is required to generalize this finding.

Overall, our results contribute to an understanding of the role of heterotypic interactions in the maturation of membraneless organelles towards the development of pathological amyloid fibrils.

## Methods

### Chemicals and reagents

ThT, rhodamine B, 1,6-hexanediol, Atto 647N NHS and polyU were purchased from Sigma-Aldrich. PolyU with a defined length of 20 nucleotides and the specific RNA were purchased from Microsynth AG. Amytracker 680 was obtained from Ebba Biotech.

### Protein purification

The hnRNPA1A sequence was ordered from Genewiz and successively cloned into a modified version of pET-15B vector including the SUMO fusion protein after the His6 tag. Briefly, BL21 DE3 cells were transformed via heat shock at 42 °C for 30 s and grown overnight at 37 °C in Luria Broth agar plates supplemented with 100 μg ampicillin ml^–1^. Cultures were scaled up in rich media, and protein expression was induced by adding 0.5 mM IPTG reagent, followed by incubation overnight at 16 °C. Cells were harvested by centrifugation, resuspended in lysis buffer (1 M NaCl, 50 mM Tris at pH 7.5, 2 mM β-mercapthoethanol), flash frozen in liquid nitrogen and stored at –80 °C. For purification, cells were thawed and supplemented with protease inhibitors (cOmplete, EDTA-free Protease Inhibitor Cocktail, Roche) and 100 μg ml^−1^ RNase A (Sigma-Aldrich). Lysis was performed by sonication, and cell debris was separated by centrifugation. Sequentially, the supernatant was transferred into a glass Becker with nickel-charged nitriloacetic acid (NTA) beads (Chelating Sepharose, GE Healthcare) and incubated for 20 min on ice. Afterwards, beads were washed three times by centrifugation with a buffer containing 1.5 M NaCl, 50 mM Tris of pH 8.5 and 50 mM imidazole to prevent nucleic acid contamination. Beads were transferred onto a gravity column, and protein elution was induced by increasing the imidazole concentration in the buffer to 500 mM. The SUMO tag was cleaved right after elution from the affinity chromatographic column by adding SUMO protease at a 1:100 ratio, and the reaction was carried out for 1 h at room temperature. Afterwards, the solution was centrifuged at 3.800*g* for 10 min to remove possible aggregates before loading on a Superdex 75 column (Cytiva) on an ÄKTA Prime system (GE Healthcare). The column was equilibrated with a buffer containing 500 mM NaCl, 50 mM Tris at pH 7.5, 10% glycerol and 2 mM 2-mercaptoethanol. Correct cleavage and purity of the eluted fractions was tested by sodium dodecyl-sulfate polyacrylamide gel electrophoresis (SDS-PAGE) coupled with Coomassie blue staining and mass spectrometry. The protein was concentrated to 300–400 μM stock solutions using concentration columns (Merck and Millipore; molecular weight cut-off (MWCO), 10 kDa). For protein labelling, protein purification was performed as explained previously, but in all buffers, Tris was substituted by phosphate-buffered saline (PBS). Pure samples were incubated overnight at 4 °C with a tenfold molar excess of Atto 647N or Atto 565 NHS-ester dye (Atto Company) previously dissolved in DMSO. Free dye was removed from the solution by size exclusion chromatography using a Superdex 75 column (Cytiva) assembled on an AKTA Explorer 100 System (GE Healthcare).

### Condensation and aggregation experiments

All the experiments were performed using 384-well plates (MatriPlate, Brooks) with a glass bottom coated with bovine serum albumin (BSA). Condensation and/or aggregation was triggered by diluting protein stock to 1:10 in buffer containing 20 mM Tris at pH 7.5 and 2 mM 2-β-mercaptoethanol unless otherwise stated, and the final reaction volume was 20 μl. For experiments over several days, wells were sealed with sticky aluminium foil (Corning) to prevent evaporation. Experiments were performed at room temperature without shaking, and no notable evaporation was observed at the end of the assay.

### PolyU labelling

RNA was labelled by conjugating Alexa Fluor TM647 Hydrazide at the 3′-hydroxyl group with periodate oxidation. Lyophilized polyU was resuspended in 100 mM sodium acetate and 2.5 mM sodium periodate solution to activate the 3′-hydroxyl group. The mixture was incubated for 50 min on ice before centrifuging and washing the activated polyU with isopropanol. The precipitated polyU was recovered in buffer with 100 mM sodium acetate, and an excess of Alexa Fluor TM647 Hydrazide (Thermo Fischer Scientific) was added to the reaction mixture. The conjugation was carried out for 24 h at 4 °C. Afterwards, the dye was removed from polyU with sequential isopropanol washes. Eventually Alexa 647 polyU was resuspended at the desired concentration in Milli-Q water and used at a 1:5 ratio with non-labelled polyU in all experiments.

### Turbidity assays

Turbidity measurements were performed using a CLARIOstar Plus plate reader (BMG Labtech), measuring the turbidity of the solution over 25 min after resuspension at a wavelength of 400 nm. The maximum absorbance value measured within 30 min was used as a proxy of dense phase volume.

### Aggregation assays

Aggregation kinetic assays were performed using ThT as the amyloid reporter, at a concentration of 20 μM. Fluorescence measurements were recorded every 15 min on a CLARIOstar Plus plate reader (BMG Labtech). Measurements were acquired using the Reader Control and MARS softwares. Samples were excited at 450 nm, and emission was recorded at 490 nm.

### Bright-field and confocal microscopy

For bright-field microscopy imaging, samples were observed with a Nikon Ti2 Eclipse inverted microscope, equipped with an LedHUB light source (Omicron), an Andor Zyla camera and a 60x oil immersion objective (Nikon; numerical aperture (NA), 1.4). Images were acquired with MicroManager software (v.2.0 gamma).

For confocal microscopy imaging, Atto 565 hnRNPA1A NHS or Atto 647N hnRNPA1A NHS was mixed with unlabelled protein at a 1:300 ratio.

To quantify droplet diameter and number, samples of hnRNPA1A at different bulk concentrations were imaged on a confocal microscope (Leica TCS SP8; Leica Application Suite X software, v.1.0) using a 63x, NA 1.4 oil objective (Leica). The microscope was equipped with a scientific complementary metal–oxide–semiconductor (sCMOS) camera (Hamamatsu ORCA-Flash4.0) and AOBS laser system.

The remaining confocal microscopy experiments were performed with a Nikon NSTORM system equipped with a re-scan confocal microscope (RCM1, Confocal·nl), an sCMOS camera (ORCA-Flash4.0 V2) and a Nikon SR Apochromat TIRF objective (100x, NA 1.49) with oil immersion. The laser excitation wavelengths were 488 nm and 647 nm for ThT and labelled protein, respectively. Image acquisition was performed using the NIS-Elements software (Nikon).

### Fluorescence-detected linear dichroism imaging

The FDLD images were acquired with the re-scan confocal microscope using a 60x, NA 1.20 oil immersion objective. To selectively change the laser polarization to either horizontally polarized or vertically polarized, a liquid crystal retarder was placed on the excitation beam path of the re-scan unit. After acquiring images with a horizontally and vertically polarized beam, the FDLD images were obtained by applying the following equation per image pixel intensity:

1 $${\mathrm{FDLD}}=\frac{{I}_{{\mathrm{V}}}+{I}_{{\mathrm{H}}}}{{I}_{{\mathrm{V}}}-{I}_{{\mathrm{H}}}}$$

where *I*_V_ and *I*_H_ are, respectively, the intensity of pixels from the image with vertically and horizontally polarized excitation. FDLD values are represented in images by applying a false-colour scale where horizontally and vertically ordered structures are respectively shown in blue and yellow, while the pixels without preferential orientation are coloured in grey.

### Fluorescence recovery after photobleaching

Data were acquired with the Leica TCS SP8 microscope described in a previous section. For this experiment, the protein bulk concentration was 20 μM and the ratio of labelled/unlabelled protein was 1:20. A region of interest with a diameter of approximately one tenth of the condensate diameter was bleached, and recovery was measured over a time frame of 30 s.

### Transmission electron microscopy

For TEM samples, 10 μl of solution was spotted on a glow discharged grid (Lacey Carbon Support Film Grids, 300 mesh, gold, Agar Scientific) for 5 min. The grid was sequentially washed with filtered, distilled water and negative stained with 2% uranyl acetate solution for 1 min. Washing and staining were repeated twice per grid. Image acquisition was performed on a TFS Morgagni 268 microscope using the iTEM 5.2 and MorgagniUI v.3 software.

### Measurements of hnRNPA1A concentration in the dilute phase

For FCS experiments, protein labelled with Atto 647 NHS was mixed with unlabelled protein at a 1:10 ratio, and samples were incubated for 72 h at room temperature prior to analysis. Labelling efficiency was measured according to the manufacturer protocol, recording the absorption spectra of the labelled protein (220–700 nm) using a Jasco V-650 spectrophotometer. FCS measurements were executed on a Leica TCS SP8 microscope equipped with a 63x, NA 1.2, W HC PL APO CS2 water immersion objective with a software-controlled correction collar. Samples were excited with a 633 nm laser at 80 MHz frequency. Emitted photons were detected using a 2 HyD (hybrid) single molecule detection detector in the range 653–703 nm, and the pinhole was set to 130 μm. The focal volume was calibrated before each experiment using a 20 nM solution of Alexa Fluor 647 Hydrazide dye (Thermo Fisher). Calibration was performed based on dye diffusion coefficient and resulted in an average focal volume of *V*_eff_ = 0.72 ± 0.02 femto liters and structural parameter *k* = 7.05 ± 0.29. The autocorrelation curves (*G*(*τ*)) were fitted with a model assuming one diffusing component without triplet contribution. The concentration of labelled protein obtained from the fitting of the autocorrelation curves was multiplied by a factor of ten to take into account the dilution factor of labelled and unlabelled protein, and divided by 0.64 to adjust for the hnRNPA1A Atto 647N labelling efficiency. Data analysis was performed using the Leica LAS X SP8 v.1.0. FCS measurements were repeated three times for three technical replicates (*n* = 9) with two independent protein preparations.

### Data analysis

Condensate number, diameter and surface/volume ratio were quantified from confocal images using a custom script in Python (v.3.9.5). The half-times of aggregation profiles were calculated from individual normalized replicates as the time needed to reach half of the maximum fluorescence intensity using a custom script in Python (v.3.9.5). FRAP experiments were analysed using a custom code in Python (v.3.9.5). Statistical analysis and data plotting were performed using R Studio (v.4.1.2). Bright-field and confocal images were analysed using Fiji. The protein disorder prediction in Fig. 1a was performed using IUPred2A (https://iupred2a.elte.hu). The secondary structure of the specific RNA with sequence 5′ CC AGC AUU AUG AAA GUG C 3′ was obtained using the RNAfold web server (http://rna.tbi.univie.ac.at/cgi-bin/RNAWebSuite/RNAfold.cgi).

### Statistics and reproducibility

All box plots report the single data points, the median of the interquartile range and the upper and lower quartile whiskers of three technical replicates unless otherwise stated in the caption of the figure.

### Reporting summary

Further information on research design is available in the Nature Portfolio Reporting Summary linked to this article.

## Online content

Any methods, additional references, Nature Portfolio reporting summaries, source data, extended data, supplementary information, acknowledgements, peer review information; details of author contributions and competing interests; and statements of data and code availability are available at 10.1038/s41557-024-01467-3.

### Supplementary information

Supplementary Information Supplementary Figs. 1–18 and Table 1.

Reporting Summary

### Source data

Source Data Fig. 1 FRAP data.

Source Data Fig. 3 ThT and turbidity assay measurements.

Source Data Fig. 4 FRAP data.

Source Data Fig. 5 ThT assay measurements.

## Data Availability

Source data are provided with this paper. The raw data of other main figures of this manuscript have been uploaded on figshare (10.6084/m9.figshare.23260172) and are publicly available.

## Code availability

All codes used in this study are deposited on figshare. The code “Droplet_diameter.py” was used to measure condensate diameters from confocal images. The code “Surface_volume_ratio.py” was used to calculate the surface to volume ratio of condensates from confocal images. The code “FRAP” was used to analyse fluorescence recovery after photobleaching. The code “Tht_kinetics.py” was used to calculate aggregation half-times of ThT assay.

## References

[1] Gebauer, F., Schwarzl, T., Valcárcel, J. & Hentze, M. W. RNA-binding proteins in human genetic disease. *Nat. Rev. Genet.* **22**, 185–198 (2021). 10.1038/s41576-020-00302-y 33235359

[2] Harrison, A. F. & Shorter, J. RNA-binding proteins with prion-like domains in health and disease. *Biochem. J.* **474**, 1417–1438 (2017). 10.1042/BCJ20160499 PMC5639257 28389532

[3] Neumann, M. et al. Ubiquitinated TDP-43 in frontotemporal lobar degeneration and amyotrophic lateral sclerosis. *Science* **314**, 130–133 (2006). 10.1126/science.1134108 17023659

[4] Rademakers, R., Neumann, M., Mackenzie, I. R. A. & Rademakers, R. TDP-43 and FUS in amyotrophic lateral sclerosis and frontotemporal dementia. *Lancet Neurol.* **9**, 995–1007 (2010). 10.1016/S1474-4422(10)70195-2 20864052

[5] Liu-Yesucevitz L (2010). Tar DNA binding protein-43 (TDP-43) associates with stress granules: analysis of cultured cells and pathological brain tissue. PLoS ONE.

[6] Wolozin, B. Regulated protein aggregation: stress granules and neurodegeneration. *Mol. Neurodegener.* **7**, 56 (2012). 10.1186/1750-1326-7-56 PMC3519755 23164372

[7] Mackenzie IR (2017). TIA1 mutations in amyotrophic lateral sclerosis and frontotemporal dementia promote phase separation and alter stress granule dynamics. Neuron.

[8] Molliex A (2015). Phase separation by low complexity domains promotes stress granule assembly and drives pathological fibrillization. Cell.

[9] Kanaan NM, Hamel C, Grabinski T, Combs B (2020). Liquid-liquid phase separation induces pathogenic tau conformations in vitro. Nat. Commun..

[10] Ray S (2020). α-Synuclein aggregation nucleates through liquid–liquid phase separation. Nat. Chem..

[11] Michaels, T. C. et al. Amyloid formation as a protein phase transition. *Nat. Rev. Phys.* **5**, 379–397 (2023).

[12] Linsenmeier M (2023). The interface of condensates of the hnRNPA1 low-complexity domain promotes formation of amyloid fibrils. Nat. Chem..

[13] Patel A (2015). A liquid-to-solid phase transition of the ALS protein FUS accelerated by disease mutation. Cell.

[14] Agarwal A, Arora L, Rai SK, Avni A, Mukhopadhyay S (2022). Spatiotemporal modulations in heterotypic condensates of prion and α-synuclein control phase transitions and amyloid conversion. Nat. Commun..

[15] Gracia P (2022). Molecular mechanism for the synchronized electrostatic coacervation and co-aggregation of alpha-synuclein and tau. Nat. Commun..

[16] Küffner AM (2021). Sequestration within biomolecular condensates inhibits Aβ-42 amyloid formation. Chem. Sci..

[17] Mathieu, C., Pappu, R. V. & Taylor, J. P. Beyond aggregation: pathological phase transitions in neurodegenerative disease. *Science* **370**, 56–60 (2020). 10.1126/science.abb8032 PMC8359821 33004511

[18] Lipiński, W. P. et al. Biomolecular condensates can both accelerate and suppress aggregation of α-synuclein. *Sci. Adv.* **8**, eabq6495 (2022). 10.1126/sciadv.abq6495 PMC10942789 36459561

[19] Lindquist S (1981). Regulation of protein synthesis during heat shock. Nature.

[20] Banerjee PR, Milin AN, Moosa MM, Onuchic PL, Deniz AA (2017). Reentrant phase transition drives dynamic substructure formation in ribonucleoprotein droplets. Angew. Chem. Int. Ed..

[21] Portz, B. & Shorter, J. Biochemical timekeeping via reentrant phase transitions. *J. Mol. Biol.* **433**, 166794 (2021). 10.1016/j.jmb.2020.166794 PMC8154630 33387533

[22] Wang C (2020). Stress induces dynamic, cytotoxicity-antagonizing TDP-43 nuclear bodies via paraspeckle LncRNA *NEAT1*-mediated liquid-liquid phase separation. Mol. Cell.

[23] Henninger JE (2021). RNA-mediated feedback control of transcriptional condensates. Cell.

[24] Maharana S (2018). RNA buffers the phase separation behavior of prion-like RNA binding proteins. Science.

[25] Kampers T, Friedhoff P, Biernat J, Mandelkow EM, Mandelkow E (1996). RNA stimulates aggregation of microtubule-associated protein tau into Alzheimer-like paired helical filaments. FEBS Lett..

[26] Nathan RD, Ralf WL, Surachai S (2003). RNA molecules stimulate prion protein conversion. Nature.

[27] Schwartz JC, Wang X, Podell ER, Cech TR (2013). RNA seeds higher-order assembly of FUS protein. Cell Rep..

[28] Zhang W (2020). Novel tau filament fold in corticobasal degeneration. Nature.

[29] Yang Y (2022). Structures of α-synuclein filaments from human brains with Lewy pathology. Nature.

[30] Arseni D (2022). Structure of pathological TDP-43 filaments from ALS with FTLD. Nature.

[31] Sharma K (2023). Cryo-EM structure of the full-length hnRNPA1 amyloid fibril. J. Mol. Biol..

[32] Kim HJ (2013). Mutations in prion-like domains in hnRNPA2B1 and hnRNPA1 cause multisystem proteinopathy and ALS. Nature.

[33] Beijer, D. et al. Characterization of *HNRNPA1* mutations defines diversity in pathogenic mechanisms and clinical presentation. *JCI Insight*10.1172/jci.insight.148363 (2021). 10.1172/jci.insight.148363 PMC8410042 34291734

[34] Lin, Y., Protter, D. S. W., Rosen, M. K. & Parker, R. Formation and maturation of phase separated liquid droplets by RNA binding proteins. *Mol. Cell* **60**, 208–219 (2015). 10.1016/j.molcel.2015.08.018 PMC4609299 26412307

[35] Tejedor AR, Garaizar A, Ramírez J, Espinosa JR (2021). Dual RNA modulation of protein mobility and stability within phase-separated condensates. Biophys. J..

[36] Tejedor AR (2022). Protein structural transitions critically transform the network connectivity and viscoelasticity of RNA-binding protein condensates but RNA can prevent it. Nat. Commun..

[37] Biancalana, M. & Koide, S. Molecular mechanism of thioflavin-T binding to amyloid fibrils. *Biochim. Biophys. Acta Proteins Proteom.* **1804**, 1405–1412 (2010). 10.1016/j.bbapap.2010.04.001 PMC2880406 20399286

[38] De Luca GM (2013). Re-scan confocal microscopy: scanning twice for better resolution. Biomed. Optics Express.

[39] Steinbach G (2019). Fluorescence-detected linear dichroism imaging in a re-scan confocal microscope equipped with differential polarization attachment. Eur. Biophys. J..

[40] Steinbach, G., Pomozi, I., Jánosa, D. P., Makovitzky, J. & Garab, G. Confocal fluorescence detected linear dichroism imaging of isolated human amyloid fibrils. Role of supercoiling. *J. Fluoresc.* **21**, 983–989 (2011). 10.1007/s10895-010-0684-3 20556489

[41] Krebs MR, Bromley EH, Donald AM (2005). The binding of thioflavin-T to amyloid fibrils: localisation and implications. J. Struct. Biol..

[42] Küffner AM (2020). Acceleration of an enzymatic reaction in liquid phase separated compartments based on intrinsically disordered protein domains. ChemSystemsChem.

[43] Capasso Palmiero U (2022). Programmable zwitterionic droplets as biomolecular sorters and model of membraneless organelles. Adv. Mater..

[44] Ritsch I (2022). Phase separation of hnRNP A1 upon specific RNA-binding observed by magnetic resonance. Angew. Chem. Int. Ed..

[45] Gruber AR, Lorenz R, Bernhart SH, Neuböck R, Hofacker IL (2008). The Vienna RNA Websuite. Nucleic Acids Res..

[46] Babinchak WM (2019). The role of liquid–liquid phase separation in aggregation of the TDP-43 low-complexity domain. J. Biol. Chem..

[47] Farag M (2022). Condensates formed by prion-like low-complexity domains have small-world network structures and interfaces defined by expanded conformations. Nat. Commun..

[48] Shen Y (2023). Solid/liquid coexistence during aging of FUS condensates. Proc. Natl Acad. Sci. USA.

[49] Emmanouilidis, L. et al. A solid beta-sheet structure is formed at the surface of FUS liquid droplets during aging. Preprint at *bioRxiv*10.1101/2023.06.02.542764 (2023). 10.1038/s41589-024-01573-w PMC11288893 38467846

[50] Pantoja-Uceda D (2021). Phe-Gly motifs drive fibrillization of TDP-43’s prion-like domain condensates. PLoS Biol..

[51] Goedert, M. et al. Assembly of microtubule-associated protein tau into Alzheimer-like filaments induced by sulphated glycosaminoglycans. *Nature* **383**, 550–553 (1996). 10.1038/383550a0 8849730

[52] Cohlberg JA, Li J, Uversky VN, Fink AL (2002). Heparin and other glycosaminoglycans stimulate the formation of amyloid fibrils from α-synuclein in vitro. Biochemistry.

[53] Rupert J, Monti M, Zacco E, Gaetano Tartaglia G (2023). A computational approach reveals the ability of amyloids to sequester RNA: the alpha synuclein case. Nucleic Acids Res..

[54] McLaurin JA, Franklin T, Zhang X, Deng J, Fraser PE (1999). Interactions of Alzheimer amyloid-β peptides with glycosaminoglycans: effects on fibril nucleation and growth. Eur. J. Biochem..

[55] Martin EW (2021). Interplay of folded domains and the disordered low-complexity domain in mediating hnRNPA1 phase separation. Nucleic Acids Res..

[56] Garcia-Pardo, J. et al. Cryo-EM structure of hnRNPDL-2 fibrils, a functional amyloid associated with limb-girdle muscular dystrophy D3. *Nat. Commun.* **14**, 239 (2023). 10.1038/s41467-023-35854-0 PMC9842712 36646699

[57] Mészáros B, Erdös G, Dosztányi Z (2018). IUPred2A: context-dependent prediction of protein disorder as a function of redox state and protein binding. Nucleic Acids Res..

