# Supplementary material for: RNA modulates hnRNPA1A amyloid formation mediated by biomolecular condensates
Source: Nat Chem. 2024 Mar 12;16(7):1052–61. doi: 10.1038/s41557-024-01467-3 (PMC11230912; doi:10.1038/s41557-024-01467-3)
Supplement: Supplementary file 1 — Supplementary Figs. 1–18 and Table 1. [file 41557_2024_1467_MOESM1_ESM.pdf]

nature chemistry

Article https://doi.org/10.1038/s41557-024-01467-3

RNA modulates hnRNPA1A amyloid formation mediated by biomolecular condensates

In the format provided by the authors and unedited

1 Table of Contents

1.1 Supplementary Figures

Supplementary Figure 1: Biochemical characterization of purified hnRNPA1A.

Supplementary Figure 2: hnRNPA1A undergoes condensation *in vitro.*

Supplementary Figure 3: The amount of the dense phase scales with protein bulk concentration.

Supplementary Figure 4: Size distribution of hnRNPA1A condensates as a function of protein bulk concentration.

Supplementary Figure 5: hnRNPA1A condensation as a function of NaCl concentration.

Supplementary Figure 6: hnRNPA1A condensation as a function of 1,6-Hexanediol concentration.

Supplementary Figure 7: Batch to batch variability in hnRNPA1A Thioflavin T (ThT) assay.

Supplementary Figure 8: Confocal images of fibrils at different hnRNPA1A bulk concentrations.

Supplementary Figure 9: hnRNPA1A forms amyloid fibrils in the absence of condensates.

Supplementary Figure 10: Formation of amyloid fibrils is promoted at the interface of hnRNPA1A condensates.

Supplementary Figure 11: Increase in the ThT fluorescence signal at the interface of the condensates is observed only with amyloid-specific dyes.

Supplementary Figure 12: ThT molecules do not exhibit alignment at the interface of the condensates in the absence of fibrils.

Supplementary Figure 13: PolyU does not show increase in ThT fluoroscence in the investigated time-frame.

Supplementary Figure 14: polyU does not form micron-sized condensates in the buffer and concentrations used in this study.

Supplementary Figure 15: polyU modulation of hnRNPA1A aggregation kinetics is independent of polyU molecular weight.

Supplementary Figure 16: First regime, bright field and fluorescence microscopy images over the reaction course.

Supplementary Figure 17: Second regime, bright field and fluorescence microscopy images over the reaction course.

Supplementary Figure 18: Third regime, bright field and fluorescence microscopy images over the reaction course.

1.2 Supplementary Tables

Supplementary Table 1: PolyU table.

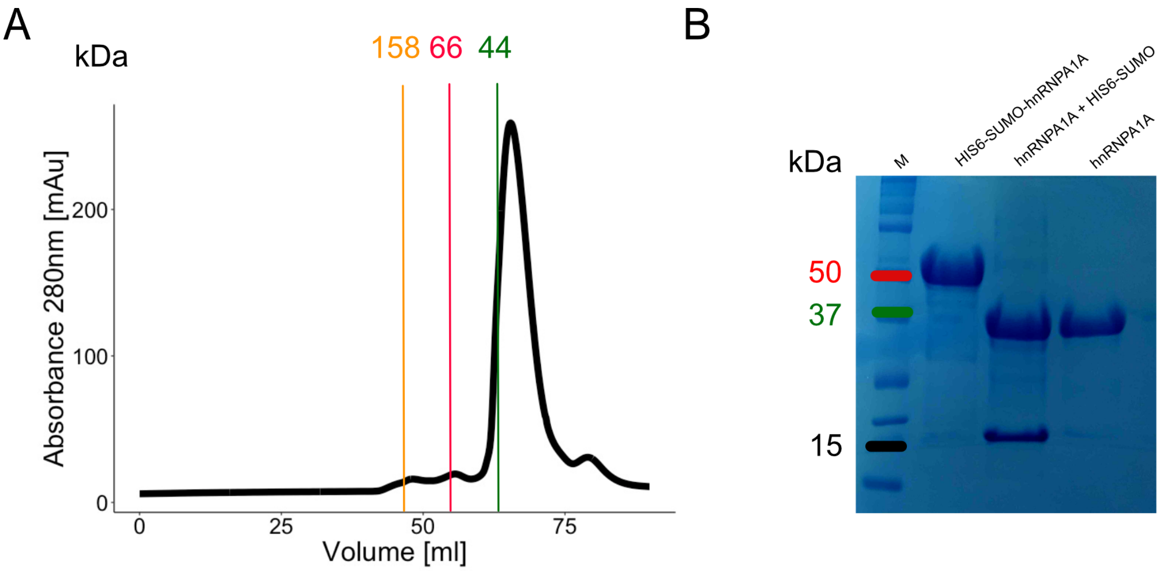

Figure 1: **Biochemical characterization of purified hnRNPA1A.** (A) Size Exclusion Chromatography (SEC) analysis of purified hnRNPA1A after cleavage of the HIS6-SUMO tag. Protein elution was monitored with UV absorbance at 280 nm. The vertical lines in the SEC chromatogram indicate the elution times corresponding to molecular weight standards of 158, 66 and 44 kDa. hnRNPA1A molecular weight is 34 kDa. (B) Correct cleavage of the HIS6-SUMO tag and purity of hnRNPA1A after SEC was confirmed by SDS page gel. M stands for marker.

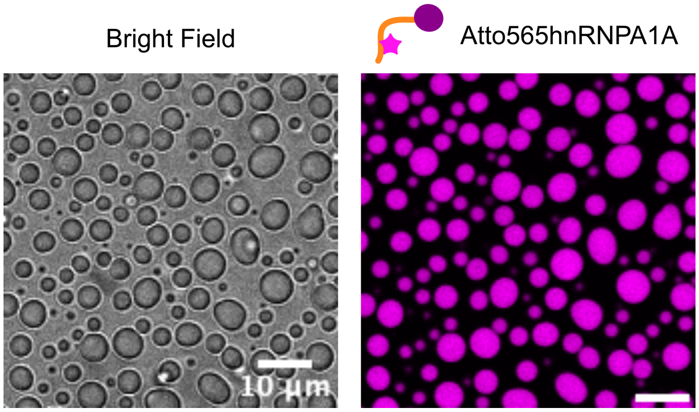

Figure 2: **hnRNPA1A undergoes condensation *in vitro*.** hnRNPA1A condensates observed with bright field (left) and fluorescent confocal (right) microscopy. Unless otherwise stated, all experiments were performed at 10 µM protein concentration in 20 mM TRIS buffer at pH 7.5 and with 2 mM β-Mercaptoethanol. Protein labelled with Atto565 NHS was diluted with unlabelled protein 1:300. Formation of hnRNPA1A protein condensates in low salt buffer was consistently observed with 10 or more different protein preparations.

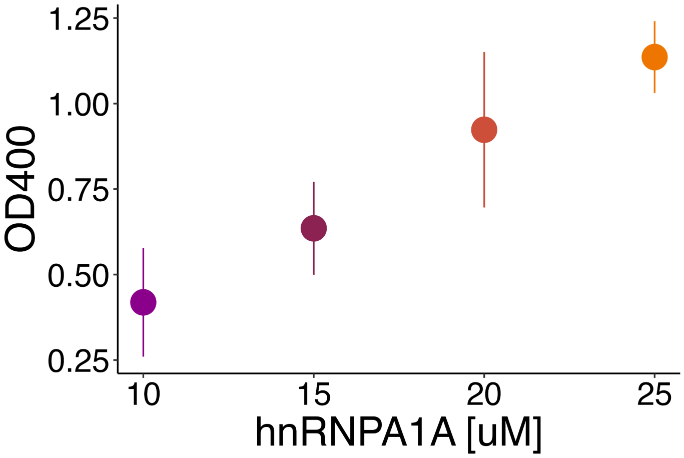

Figure 3: **The amount of the dense phase scales with protein bulk concentration.** Increasing bulk concentration of hnRNPA1A in solution results in higher turbidity of the solution, indicating an increase of the amount of the dense phase. The experiment was performed in technical replicates and on two distinct protein preparations. Data points are the mean and error bars show standard deviations of technical triplicates.

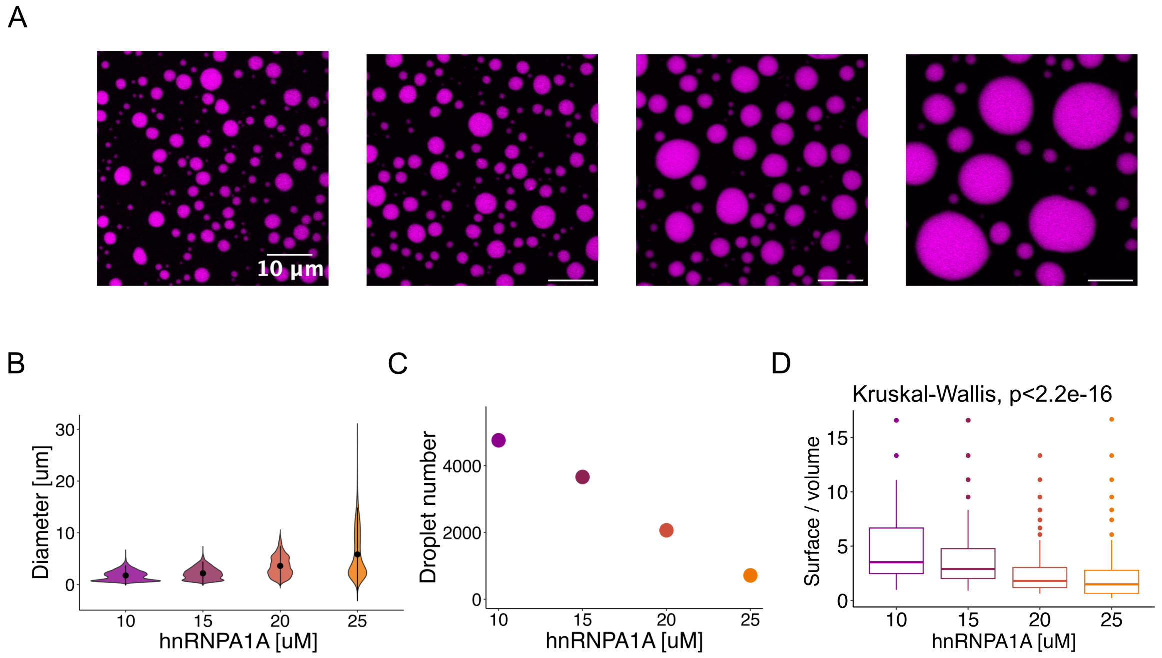

Figure 4: **Size distribution of hnRNPA1A condensates as a function of protein bulk concentration.** (A) Representative confocal images of hnRNPA1A condensates at different protein bulk concentrations (from left to right, 10 µM, 15 µM, 20 µM and 25 µM). A total of 10 confocal images, 5 from each technical duplicate, were analyzed to measure the diameter of condensates for the conditions tested. The experiment was performed for two distinct protein preparations yielding similar results. (B) Violin plot reporting the median and the kernel probability density of the diameter of hnRNPA1A condensates at different bulk protein concentration. Wiskers represents the interquartile range. (C) Number of condensates analyzed at each protein bulk concentration. Each data point corresponds to the sample size (n-value) of the conditions studied (10 µM n= 4761, 15 µM n= 3663, 20 µM n= 2066 and 25 µM n=715) (D) The box blots report the median of the surface to volume ratio calculated from each condition with respective interquartile range and the upper and lower quartile whiskers. Dots represent the outliers. Although differences among average values are significant (Kruskas-Wallis, $p - value \leq 2.2 * 10^{-16}$), there is a great overlapping of the distributions among the different samples. Moreover, we note the presence of several outliers that do not influence ranked-based statistical tests [1].

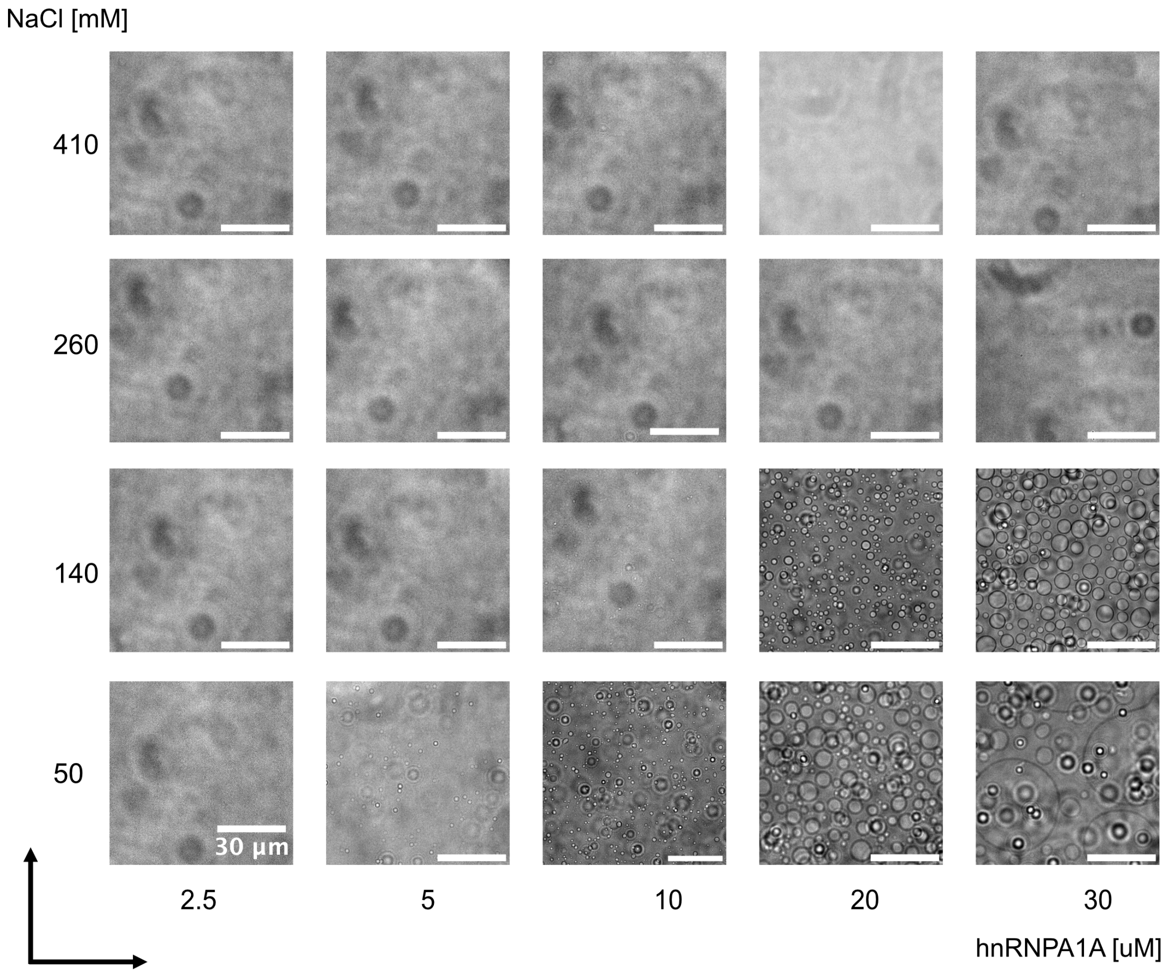

Figure 5: **hnRNPA1A condensation as a function of NaCl concentration**. Absence or presence of condensation was evaluated from the appearance of micron-sized droplets using bright field microscopy. The experiment was performed for two distinct protein preparations yielding similar results.

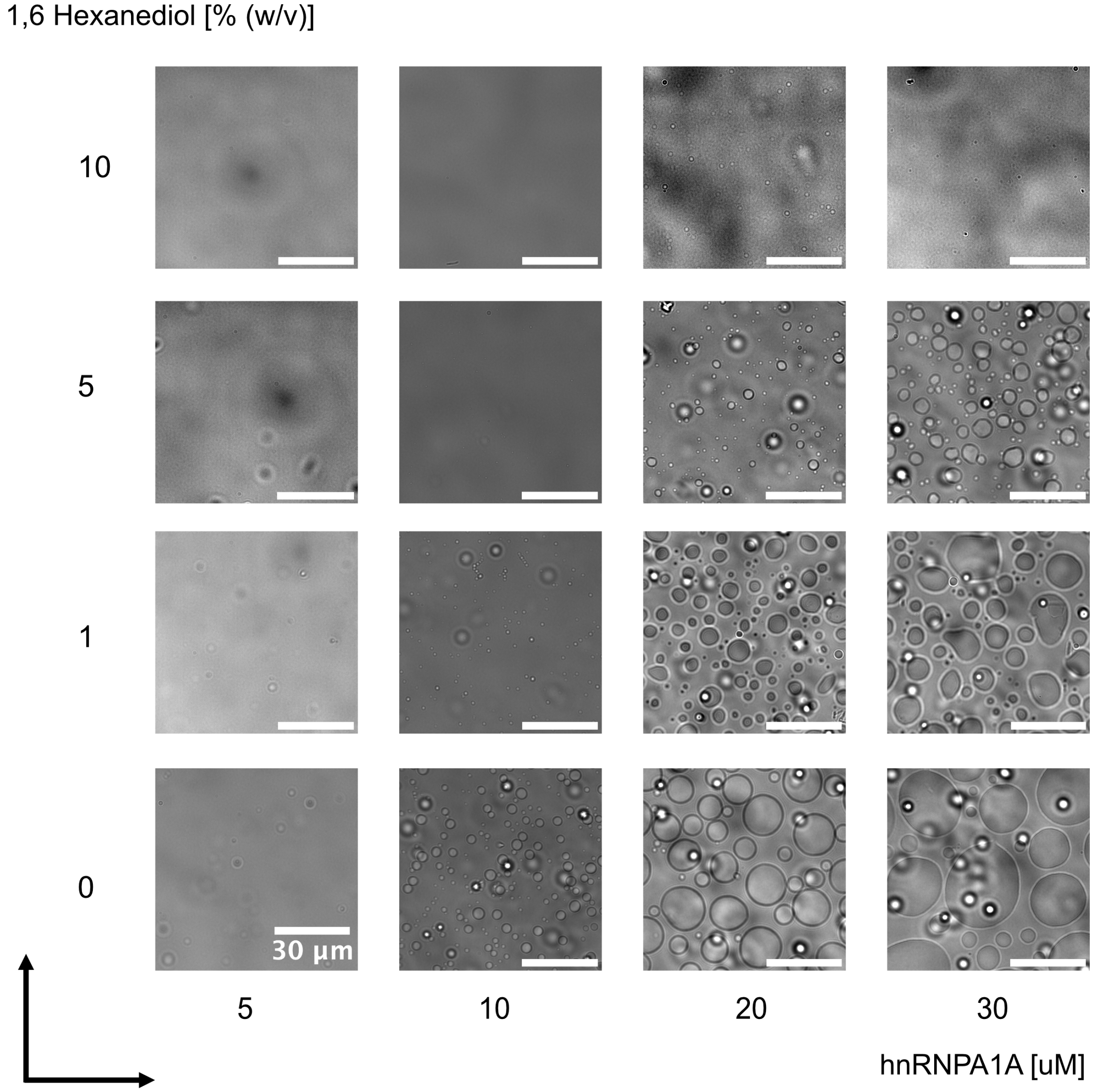

Figure 6: **hnRNPA1A condensation as a function of 1,6-Hexanediol concentration.** Absence or presence of condensation was evaluated from the appearance of micron-sized droplets using bright field microscopy. The experiment was performed for two distinct protein preparations yielding similar results.

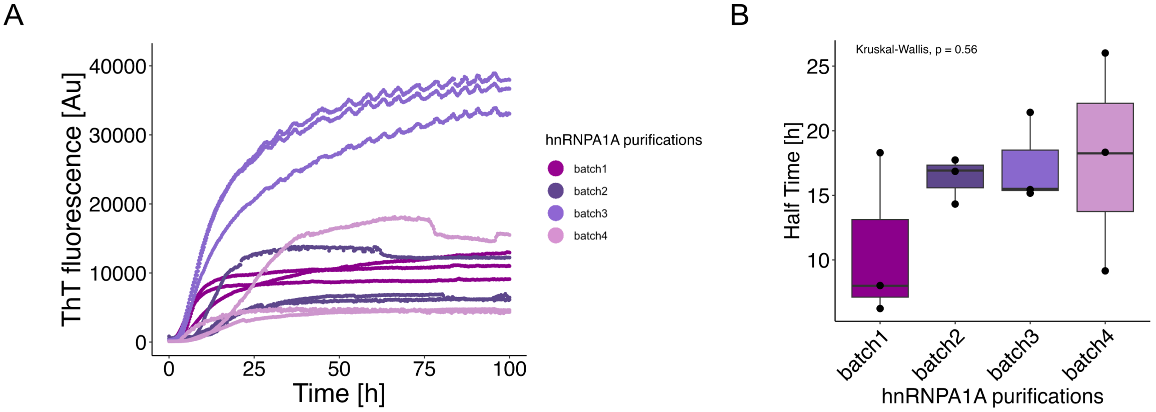

Figure 7: **Batch to batch variability in hnRNPA1A Thioflavin T (ThT) assay.** (A) Aggregation curves of technical triplicates from different hnRNPA1A batches at 10 µM concentration. Fluorescence ThT endpoints varied from batch to batch, and often we observed curves with a steep increase of ThT intensity in the first 24 hours followed by a slow increasing plateau.(B) The box blots report the half times calculated from each curve (black dots), the median half time, the interquartile range and the upper and lower quartile whiskers. The median of half times from technical replicates varied from 10 to 17 hours across protein batches. Nevertheless, non-paramteric Kruscal-Wallis test shows no significant difference between aggregation times among different protein purifications ($p - value = 0.56$) (n=3). This data show our batch to batch time-scale variability, and the number of times the experiment was repeated corresponds to the number of batches (4 different protein batches).

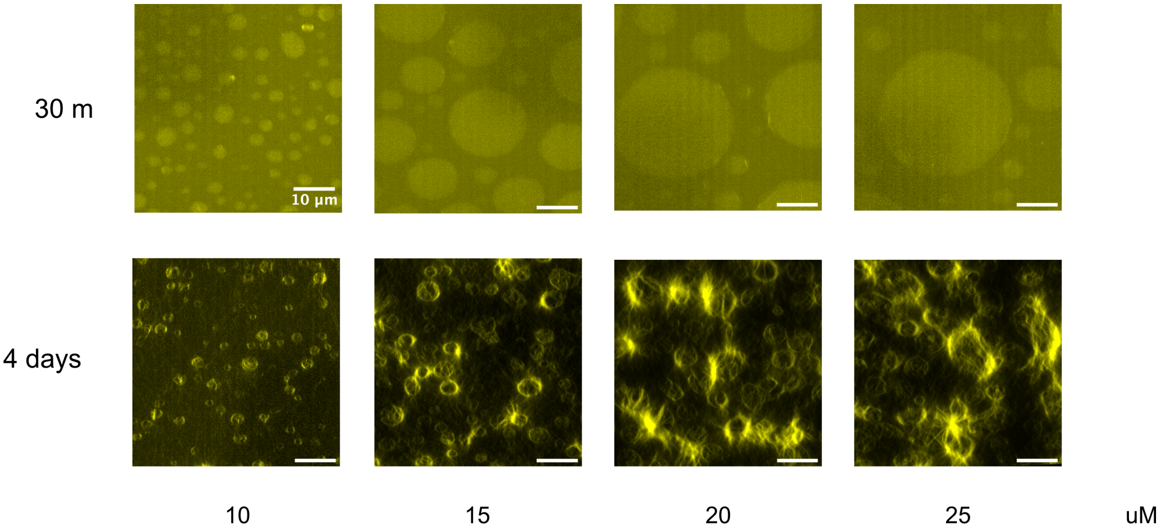

Figure 8: **Confocal images of fibrils at different hnRNPA1A bulk concentrations.** Confocal images of hnRNPA1A condensates at different bulk concentrations (10, 15, 20 and 25 µM) stained with ThT. In the top row we show condensates at 30 minutes of incubation. In the bottom row we show condensates after 4 days of incubation at room temperature. The experiment was repeated with three different protein preparations.

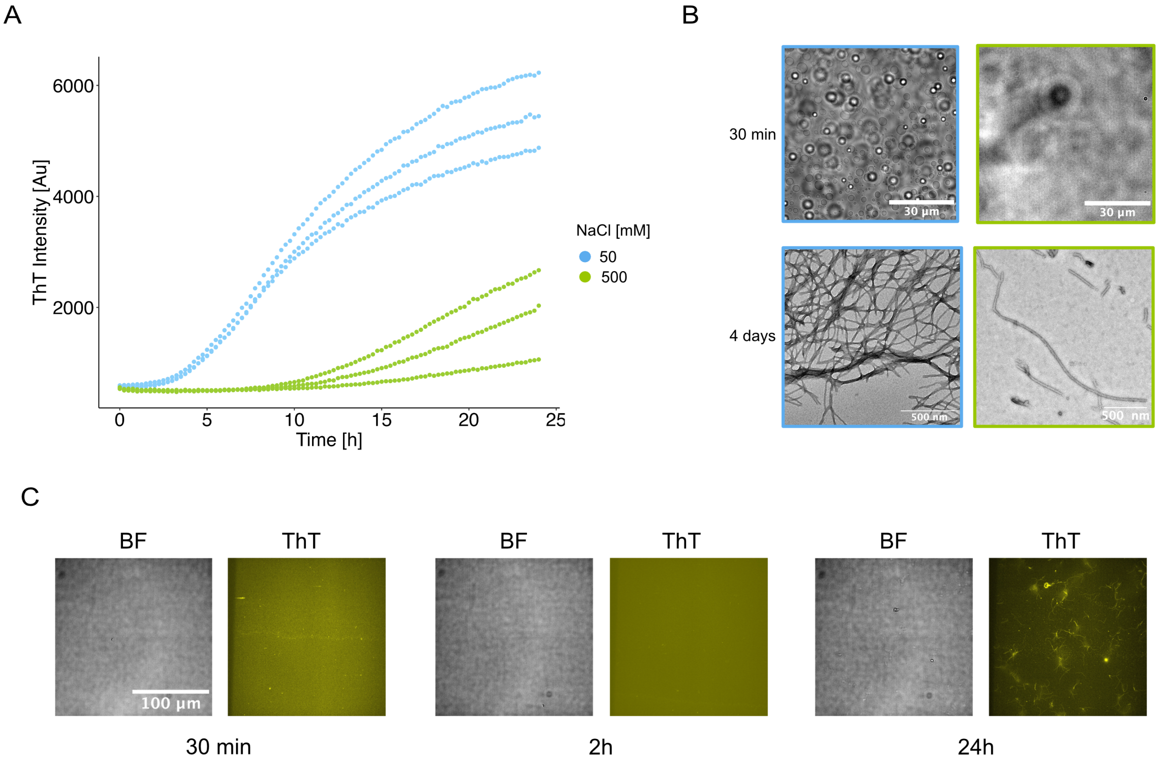

Figure 9: **hnRNPA1A forms amyloid fibrils in the absence of condensates.** (A) ThT aggregation assays of hnRNPA1A in presence (light-blue curve) and absence (green curve) of condensates. Formation of condensates was suppressed by increasing NaCl concentration above 500 mM. The graph shows three technical replicates per condition. The experiment was performed on at least two distinct protein preparations. (B) Top row: bright field microscopy images showing presence and absence of condensation at low (light-blue) and high salt (green) concentration, respectively. The experiment was repeated with at least three different protein preparations. Bottom row: TEM micrographs showing formation of amyloid fibrils at the end of aggregation reaction in the two conditions tested. TEM imaging was performed on one protein batch. (C) Bright field and fluorescence microscopy images showing absence of condensates in presence of 500 mM NaCl and at different time points during hnRNPA1A aggregation. The experiment was repeated with two distinct protein preparations.

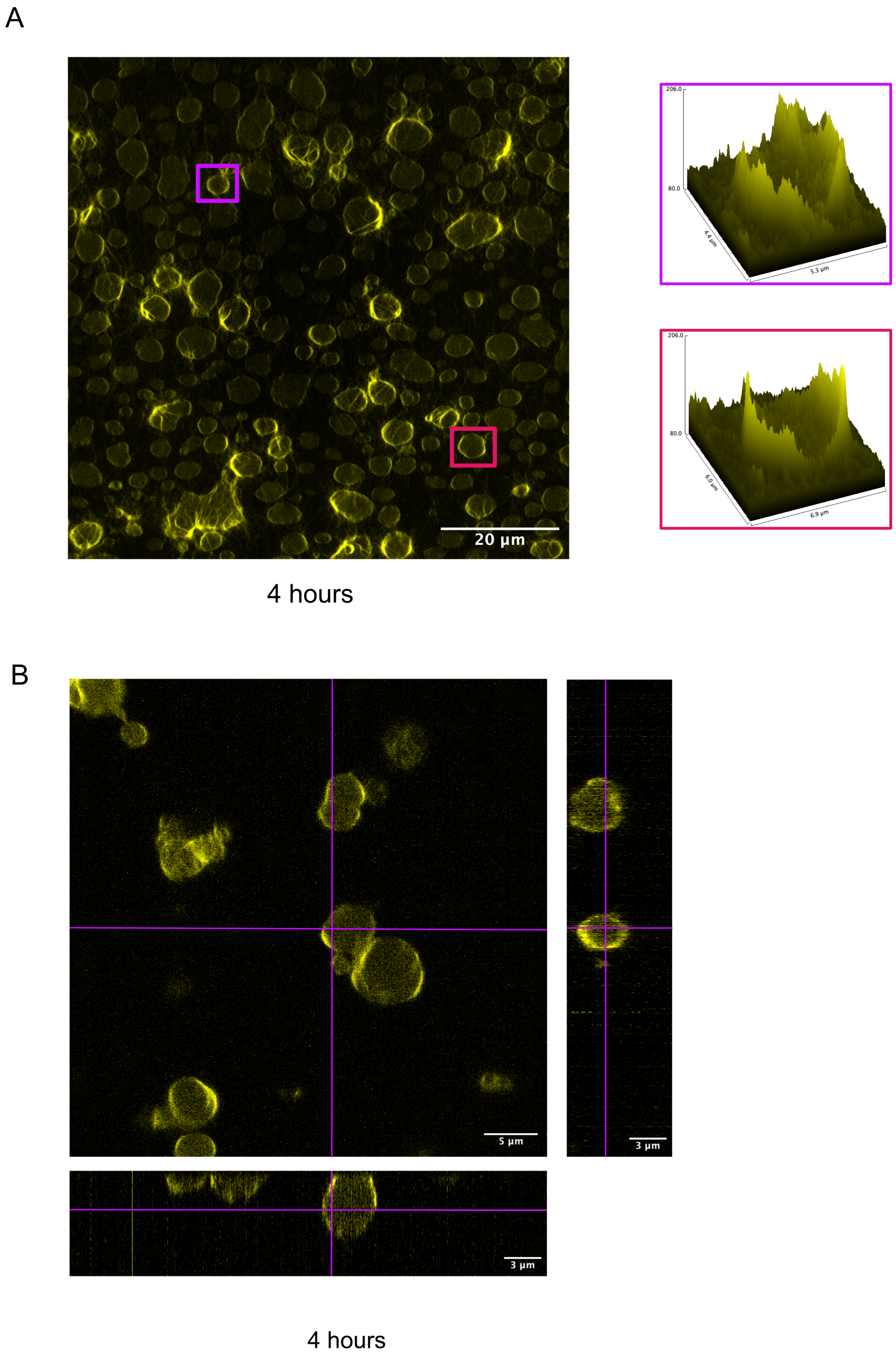

Figure 10: **Formation of amyloid fibrils is promoted at the interface of hnRNPA1A condensates.** (A) Large view of hnRNPA1A condensates at 4 hours of incubation. The image shows the presence of a ThT-positive rim. Next to the image, the intensity profiles corresponding to the areas enclosed within the purple and magenta boxes are shown. (B) Orthogonal view of a z-stack of hnRNPA1A condensates in the presence of ThT after 4 hours of incubation. Confocal z-stacks of hnRNPA1A condensates in presence of ThioflavinT were acquired for one single protein preparation.

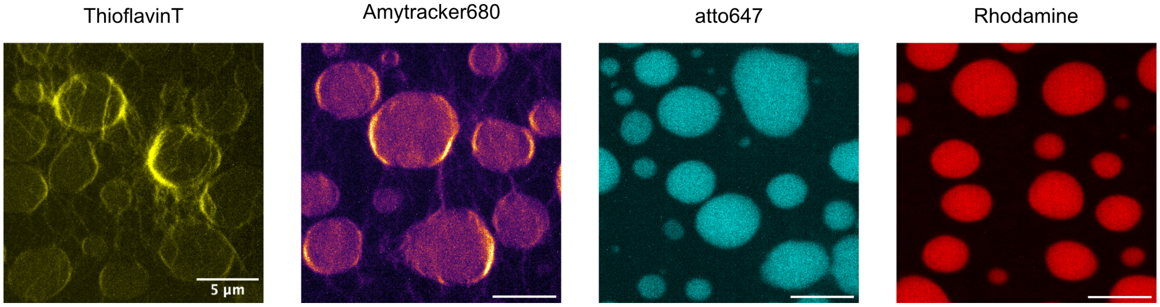

Figure 11: **Increase in the ThT fluorescence signal at the interface of the condensates is observed only with amyloid-specific dyes.** Confocal images of hnRNPA1A condensates taken after 4 hours of incubation in the presence of different dyes (ThT , Amytracker680, Atto647 and Rhodamine). We observe increased fluorescence at the interface of the condensates only in presence of dyes known to report on the presence of amyloid fibrils (ThT and Amytracker680). This experiment was performed in technical duplicates for one single protein batch.

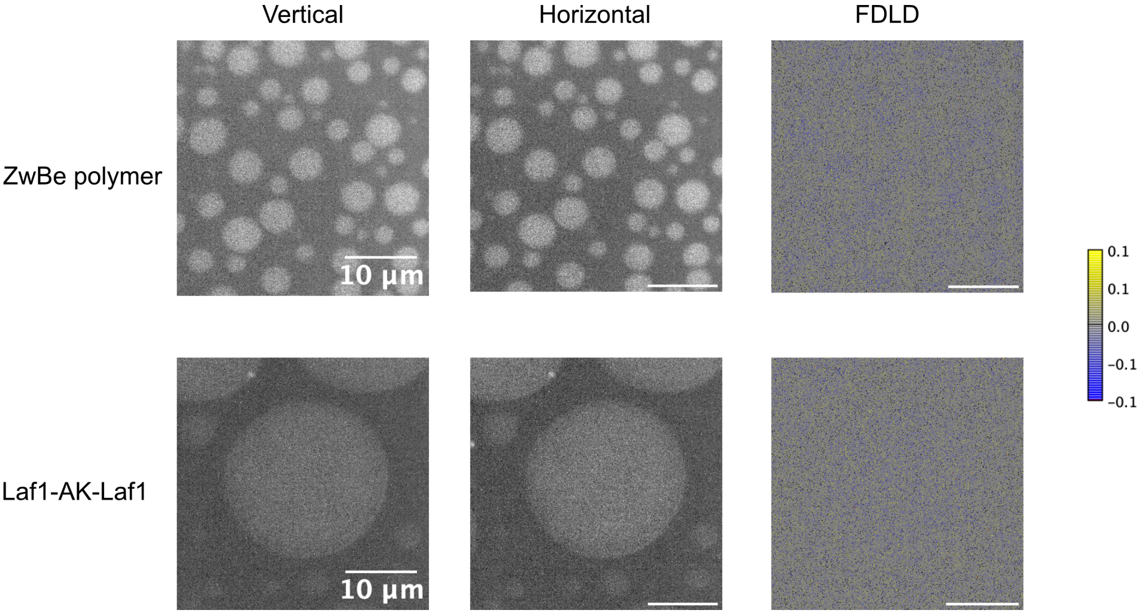

Figure 12: **ThT molecules do not exhibit alignment at the interface of the condensates in the absence of fibrils.** Preferential orientation of ThT molecules was not observed at the interface of laf1-AK-laf1 condensates [2], or of zwitterionic polymeric coacervates [3], proving that the observed effect is related to the presence of fibrils at the interface of hnRNPA1A coacervates. This experiment was performed in technical duplicates for one single protein and polymer batches.

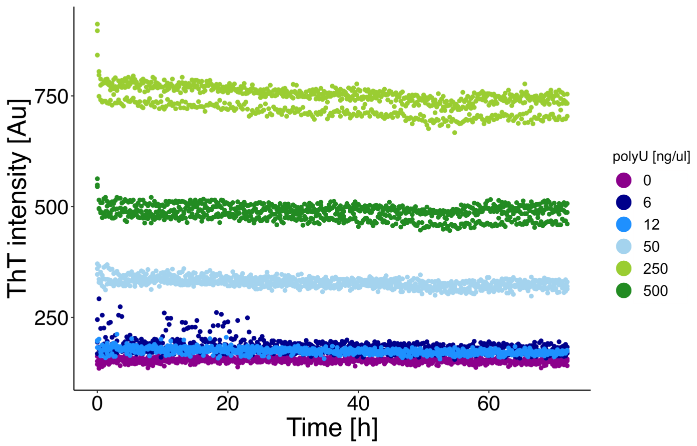

Figure 13: **PolyU does not show increase in ThT fluoroscence in the investigated time-frame.** ThT fluorescence intensity of solutions with different polyU concentrations over time. Presence of RNA in solution has a negligible effect on ThT fluorescence intensity compared to the endpoint values of hnRNPA1A aggregation assays. Moreover, the signal does not change over time. This experiment was repeated independently twice.

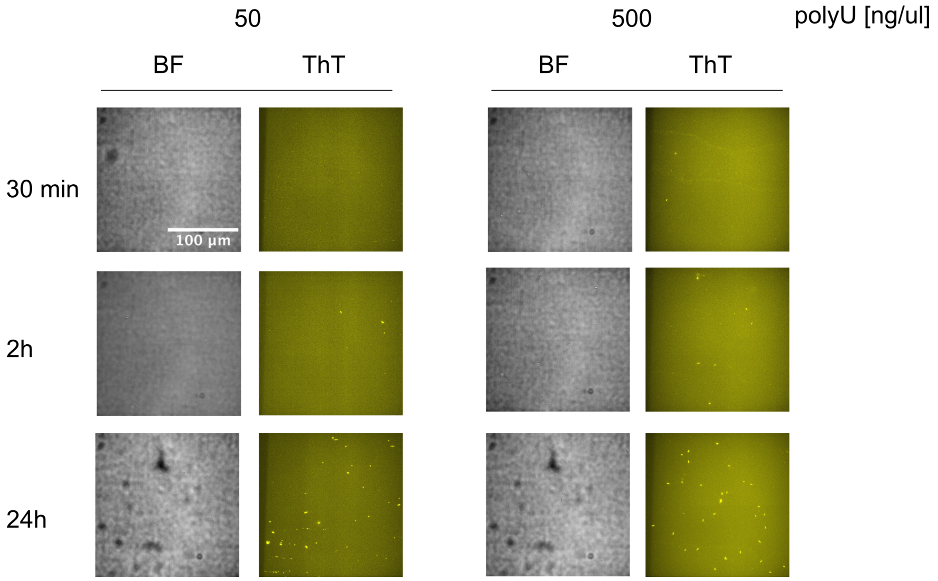

Figure 14: **polyU does not form micron-sized condensates in the buffer and concentrations used in this study** Bright field and fluorescence microscopy images of polyU solutions with ThT at the concentrations used in this article. The micrographs show that polyU does not undergo condensation under the conditions investigated in this work. The experiment was performed one time in technical duplicates.

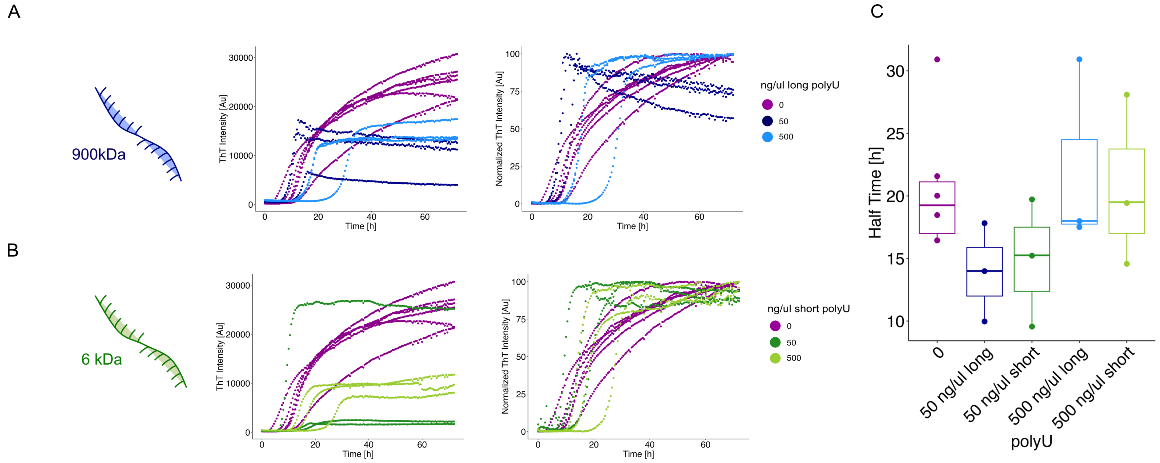

Figure 15: **polyU modulation of hnRNPA1A aggregation kinetics is independent of polyU molecular weight.** (A-B) Raw and normalized ThT aggregation assays of 10 uM hnRNAP1A in the absence and presence of different concentrations of long (A) and short (B) polyU. (C) Aggregation half times from normalized ThT profiles show how polyU modulation of hnRNPA1A aggregation rate is independent of RNA molecular weight. All experiments in this figure were performed with three technical replicates and biological duplicates.

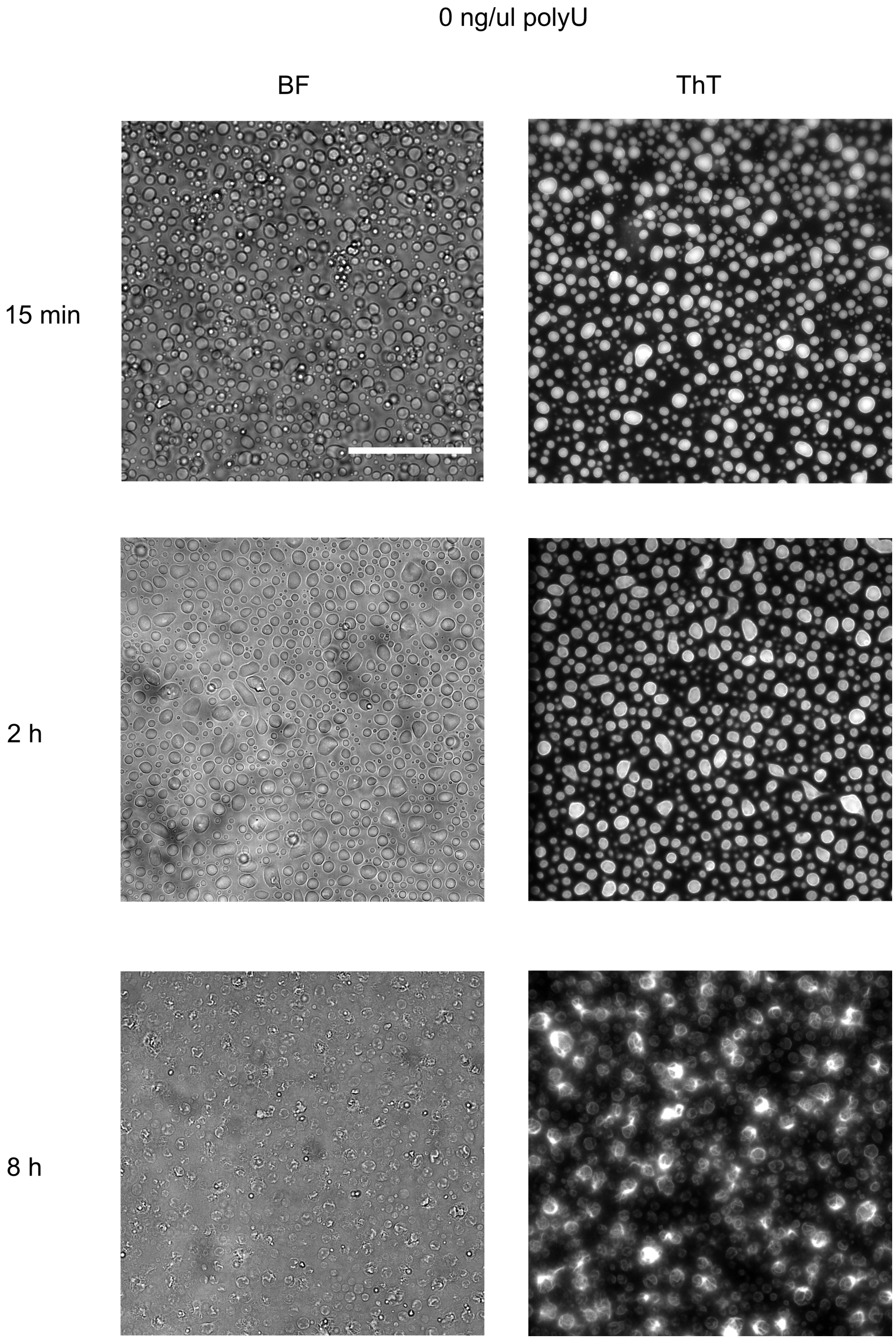

Figure 16: **First regime, bright field and fluorescence microscopy images over the reaction course.** Aggregation reaction of hnRNPA1A followed by bright field and fluorescence microscopy during the first 8 hours in absence of polyU. ThT was used as amyloid fibril reporter, and scale bar is 50 µm. This experiment was performed on one protein batch.

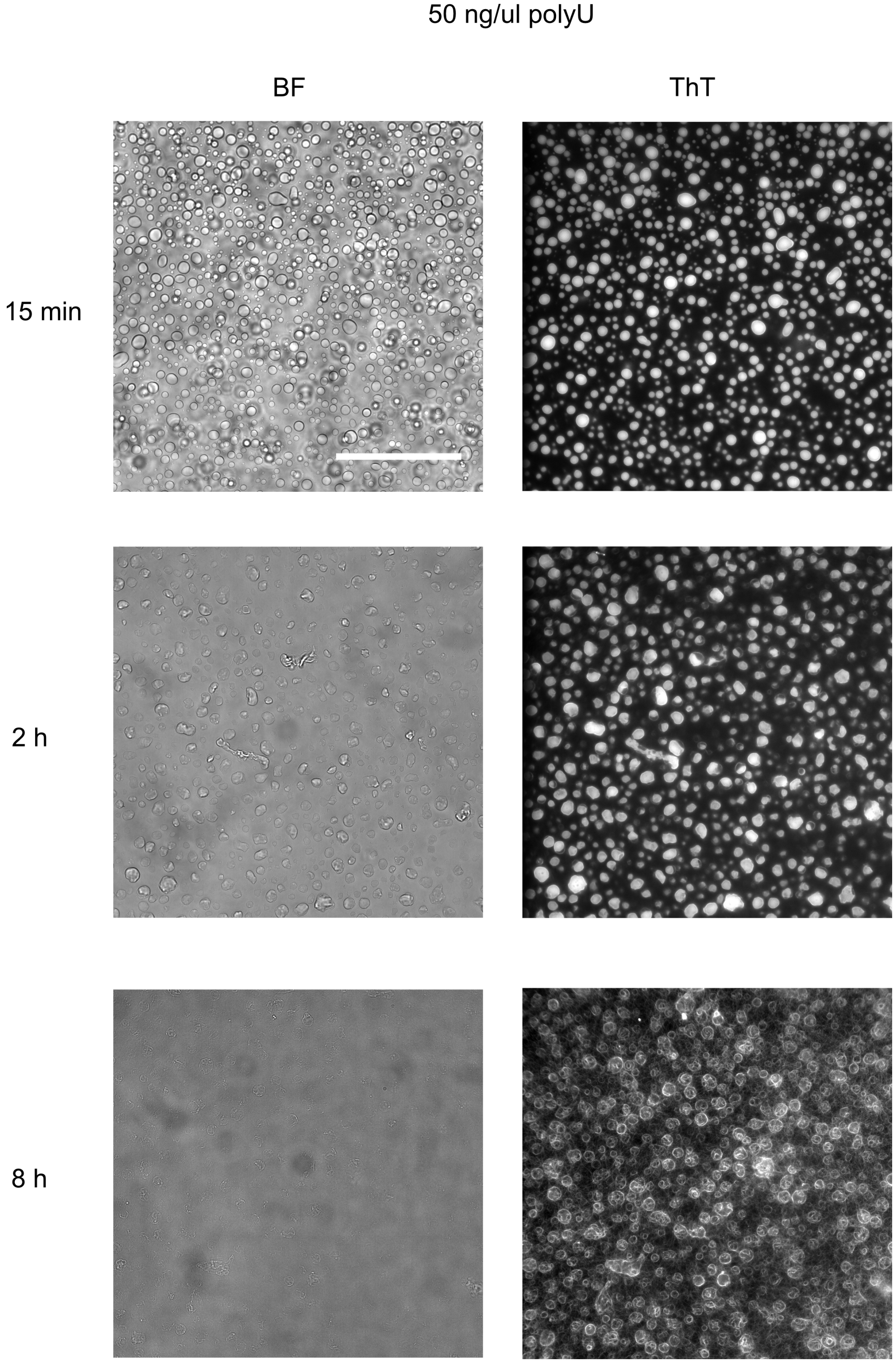

Figure 17: **Second regime, bright field and fluorescence microscopy images over the reaction course.** Aggregation reaction of hnRNPA1A followed by bright field and fluorescence microscopy during the first 8 hours in presence of 50 ng/µl of polyU. ThT was used as amyloid fibril reporter, and scale bar is 50 µm. This experiment was performed on one protein batch.

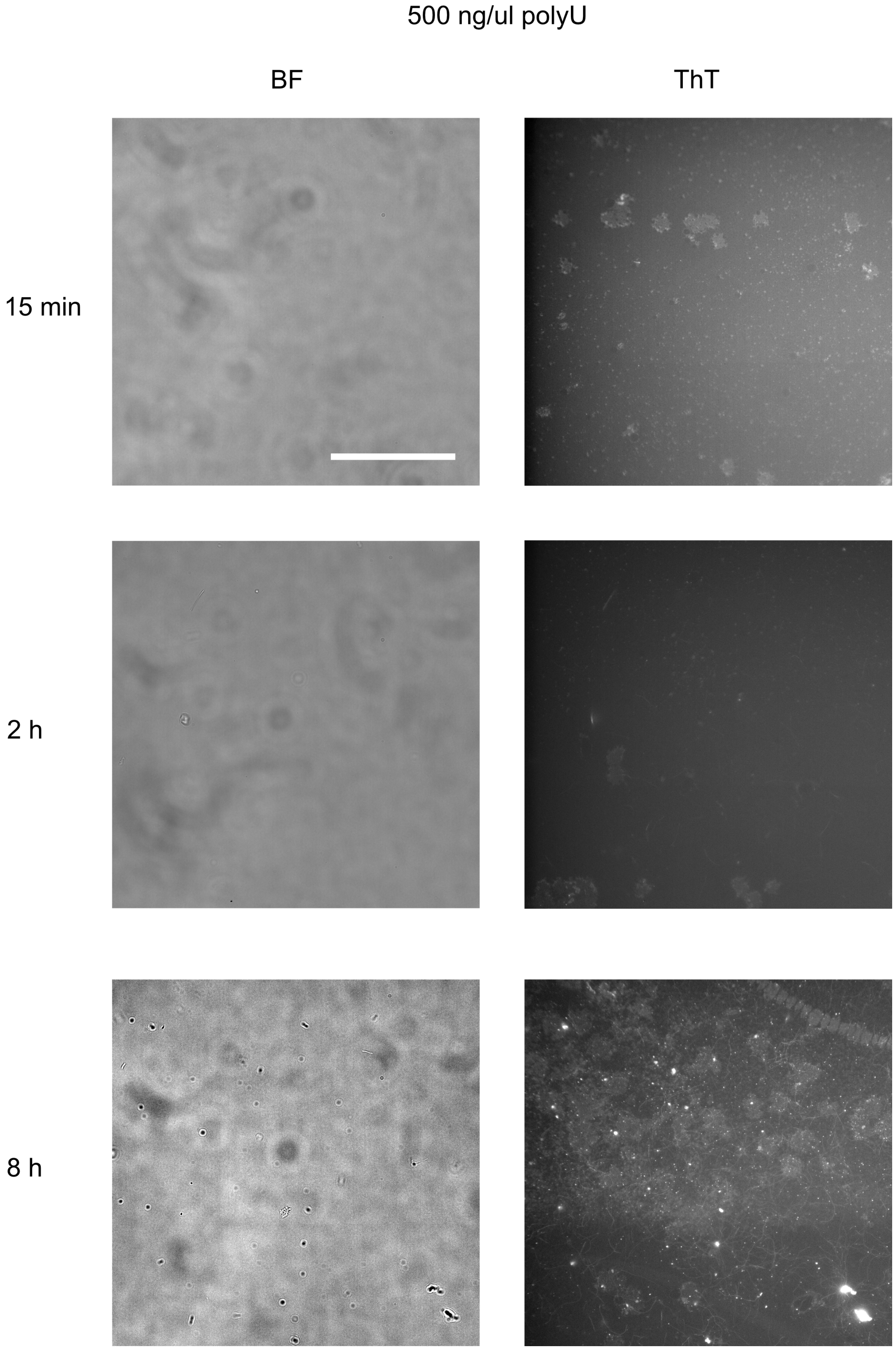

Figure 18: **Third regime, bright field and fluorescence microscopy images over the reaction course.** Aggregation reaction of hnRNPA1A followed by bright field and fluorescence microscopy during the first 8 hours in presence of 500 ng/µl of polyU. ThT was used as amyloid fibril reporter, and scale bar is 50 µm. This experiment was performed on one protein batch.

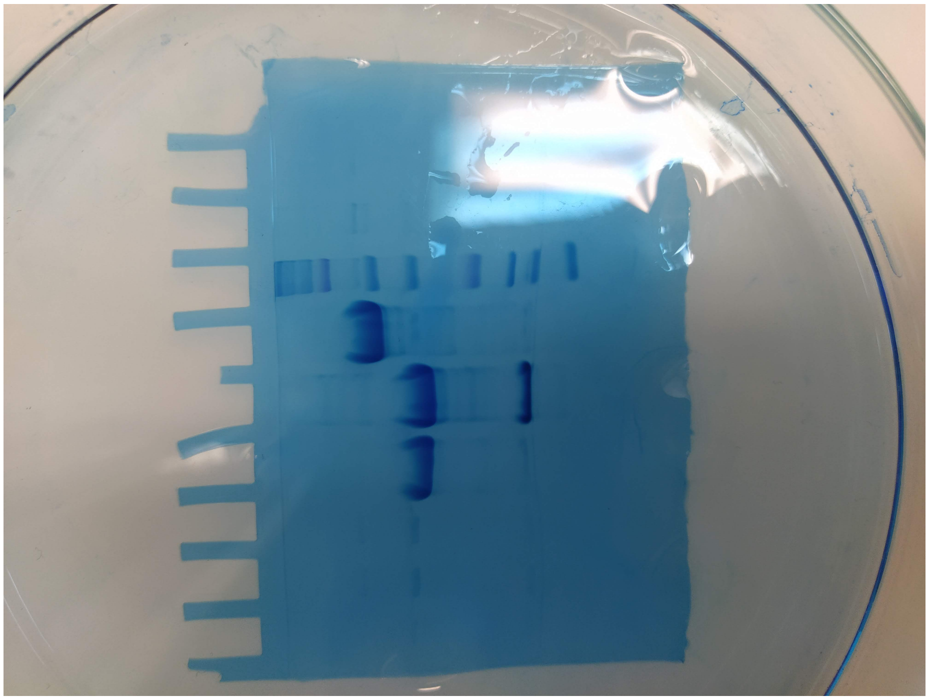

Figure 19: Uncropped gel image in Supplementary Figure 1.

polyU molecular weight: 900 kDa
UMP molecular weight: 324 Da
number of UMP per molecule : 2776
polyU net charge pH 7.5 : -2776
hnRNPA1A net charge pH 7.5 : +6

polyU [ng/ul]	polyU uM	Protein:RNA molar ratio	Charge ratio
3	0.003	3000	6.48
6	0.007	1500	3.24
12	0.013	750	1.62
25	0.028	360	0.78
50	0.056	180	0.39
100	0.111	90	0.19
250	0.278	36	0.09

Table 1: **PolyU table.** The long polyU used in this article has a poly-disperse molecular weight ranging from 800 to 1000 kDa. As an average molecular weight we considered 900 kDa, and the average net charge was estimated from the number of uridine monophospates in one molecule. To this aim, the average molecular weight of polyU was divided by the molecular weight of one UMP molecule (900.000 / 324.2 = 2776).

References

[1] F. S. Nahm, "Nonparametric statistical tests for the continuous data: The basic concept and the practical use," *Korean Journal of Anesthesiology*, vol. 69, no. 1, pp. 8–14, Feb. 2016, ISSN: 20057563. DOI: 10.4097/kjae.2016.69.1.8.

[2] A. M. Küffner, M. Prodan, R. Zuccarini, U. Capasso Palmiero, L. Faltova, and P. Arosio, "Acceleration of an Enzymatic Reaction in Liquid Phase Separated Compartments Based on Intrinsically Disordered Protein Domains," *ChemSystemsChem*, vol. 2, no. 4, Jul. 2020, ISSN: 2570-4206. DOI: 10.1002/syst.202000001.

[3] U. Capasso Palmiero, C. Paganini, M. R. Kopp, M. Linsenmeier, A. M. Küffner, and P. Arosio, "Programmable Zwitterionic Droplets as Biomolecular Sorters and Model of Membraneless Organelles," *Advanced Materials*, vol. 34, no. 4, Jan. 2022, ISSN: 15214095. DOI: 10.1002/adma.202104837.
