# Supplementary material for: RNA modulates hnRNPA1A amyloid formation mediated by biomolecular condensates
Source: Nat Chem. 2024 Mar 12;16(7):1052–61. doi: 10.1038/s41557-024-01467-3 (PMC11230912; doi:10.1038/s41557-024-01467-3)
Supplement: Supplementary file 2 — Reporting Summary [file 41557_2024_1467_MOESM2_ESM.pdf]

nature portfolio

Corresponding author(s): Paolo Arosio

Last updated by author(s): 30.01.2024

Reporting Summary

Nature Portfolio wishes to improve the reproducibility of the work that we publish. This form provides structure for consistency and transparency in reporting. For further information on Nature Portfolio policies, see our Editorial Policies and the Editorial Policy Checklist.

Statistics

For all statistical analyses, confirm that the following items are present in the figure legend, table legend, main text, or Methods section.

n/a	Confirmed	
☐	☒	The exact sample size (*n*) for each experimental group/condition, given as a discrete number and unit of measurement
☐	☒	A statement on whether measurements were taken from distinct samples or whether the same sample was measured repeatedly
☐	☒	The statistical test(s) used AND whether they are one- or two-sided<br>*Only common tests should be described solely by name; describe more complex techniques in the Methods section.*
☒	☐	A description of all covariates tested
☐	☒	A description of any assumptions or corrections, such as tests of normality and adjustment for multiple comparisons
☐	☒	A full description of the statistical parameters including central tendency (e.g. means) or other basic estimates (e.g. regression coefficient) AND variation (e.g. standard deviation) or associated estimates of uncertainty (e.g. confidence intervals)
☐	☒	For null hypothesis testing, the test statistic (e.g. *F*, *t*, *r*) with confidence intervals, effect sizes, degrees of freedom and *P* value noted<br>*Give P values as exact values whenever suitable.*
☒	☐	For Bayesian analysis, information on the choice of priors and Markov chain Monte Carlo settings
☒	☐	For hierarchical and complex designs, identification of the appropriate level for tests and full reporting of outcomes
☒	☐	Estimates of effect sizes (e.g. Cohen's *d*, Pearson's *r*), indicating how they were calculated

Our web collection on statistics for biologists contains articles on many of the points above.

Software and code

Policy information about availability of computer code

Data collection	Bright field microscopy: MicroManager software (version 2.0 gamma)<br>Re-scan confocal microscopy: NIS Elements 5.20 Advanced research software (Nikon)<br>Confocal microscopy: Leica LAS X SP8 software (version 1.0)<br>Plate reader for ThioflavinT assays: Reader Control and MARS software (BMG Labtech)<br>Fluoresce Correlation Spectroscopy: Leica LAS X SP8 Version 1.0<br>TEM Morgagni: iTEM 5.2 and MorgagniUI Version 3
Data analysis	Droplets diameter, number, surface/volume ratio : in-house written programs in Python (version 3.9.5)<br>Image Analysis and FDLD calculation: Fiji 2.9.0<br>All data were plotted using R studio 4.1.2<br>Protein disorder prediction: IU pred https://iupred2a.elte.hu<br>RNA secondary structure prediction: RNA fold WebServer http://rna.tbi.univie.ac.at//cgi-bin/RNAWebSuite/RNAfold.cgi?PAGE=3&ID=XS3fKUfcoq<br>Fluoresce Correlation Spectroscopy: Leica LAS X SP8 Version 1.0

For manuscripts utilizing custom algorithms or software that are central to the research but not yet described in published literature, software must be made available to editors and reviewers. We strongly encourage code deposition in a community repository (e.g. GitHub). See the Nature Portfolio guidelines for submitting code & software for further information.

Data

Policy information about availability of data

All manuscripts must include a data availability statement. This statement should provide the following information, where applicable:

- Accession codes, unique identifiers, or web links for publicly available datasets
- A description of any restrictions on data availability
- For clinical datasets or third party data, please ensure that the statement adheres to our policy

Measurements represented in the graphs in Figure 1 (FRAP experiments) Figure 3 (ThT aggregation assays, turbidity assays), Figure 4 (FRAP experiments) and Figure 5 (ThT aggregation assays) are reported as Source Data. The raw data of all the other the main figures of this manuscript have been uploaded on Figshare and are publicly available. IUpred2A (https://iupred2a.elte.hu) was used for analysis of hnRNPA1A to predict disordered regions. RNAfold webserver (http://rna.tbi.univie.ac.at//cgi-bin/RNAWebSuite/RNAfold.cgi0) was used for the prediction of the secondary structure of the specific RNA (http://rna.tbi.univie.ac.at/cgi-bin/RNAWebSuite/RNAfold.cgi).

Human research participants

Policy information about studies involving human research participants and Sex and Gender in Research.

Reporting on sex and gender	n/a
Population characteristics	n/a
Recruitment	n/a
Ethics oversight	n/a

Note that full information on the approval of the study protocol must also be provided in the manuscript.

Field-specific reporting

Please select the one below that is the best fit for your research. If you are not sure, read the appropriate sections before making your selection.

☒ Life sciences ☐ Behavioural & social sciences ☐ Ecological, evolutionary & environmental sciences

For a reference copy of the document with all sections, see nature.com/documents/nr-reporting-summary-flat.pdf

Life sciences study design

All studies must disclose on these points even when the disclosure is negative.

Sample size	No sample size calculation was perform. Each data point represent three independent measurements.
Data exclusions	No data was excluded.
Replication	Experiments were executed in triplicates and with independent protein batches. We observed time-scale variations in between different proteins batches, but the relative trends and major observations remained consistent in all experiments.
Randomization	There was not group randomization.
Blinding	Experiments were not blinded.

Reporting for specific materials, systems and methods

We require information from authors about some types of materials, experimental systems and methods used in many studies. Here, indicate whether each material, system or method listed is relevant to your study. If you are not sure if a list item applies to your research, read the appropriate section before selecting a response.

Materials & experimental systems

n/a	Involved in the study
☒	☐ Antibodies
☒	☐ Eukaryotic cell lines
☒	☐ Palaeontology and archaeology
☒	☐ Animals and other organisms
☒	☐ Clinical data
☒	☐ Dual use research of concern

Methods

n/a	Involved in the study
☒	☐ ChIP-seq
☒	☐ Flow cytometry
☒	☐ MRI-based neuroimaging
